# Supplementary figures and images for: Efficient consideration of coordinated water molecules improves computational protein-protein and protein-ligand docking discrimination
Source: PLoS Comput Biol. 2020 Sep 21;16(9):e1008103. doi: 10.1371/journal.pcbi.1008103 (PMC7529342; doi:10.1371/journal.pcbi.1008103)

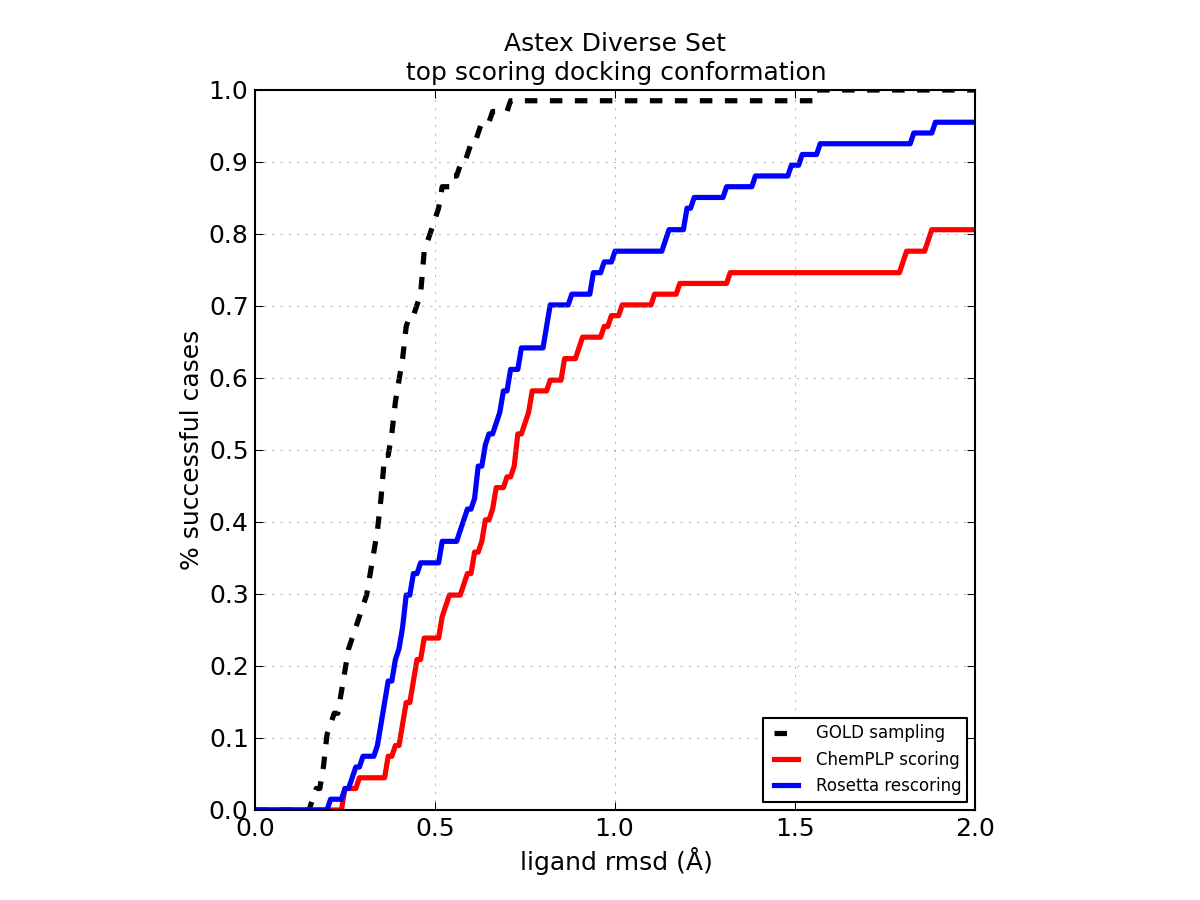

Supplement: S1 Fig — Results for rescoring Astex Diverse Set. Docking conformations initially generated and scored by GOLD (red) were rescored with the Rosetta REF2015 energy function (blue). The theoretical scoring success is determined by the initial GOLD sampling (black dashed) for the 67 cases of the Astex Diverse Set that do not coordinate an ion in the binding site. (PNG) [file pcbi.1008103.s002.png]

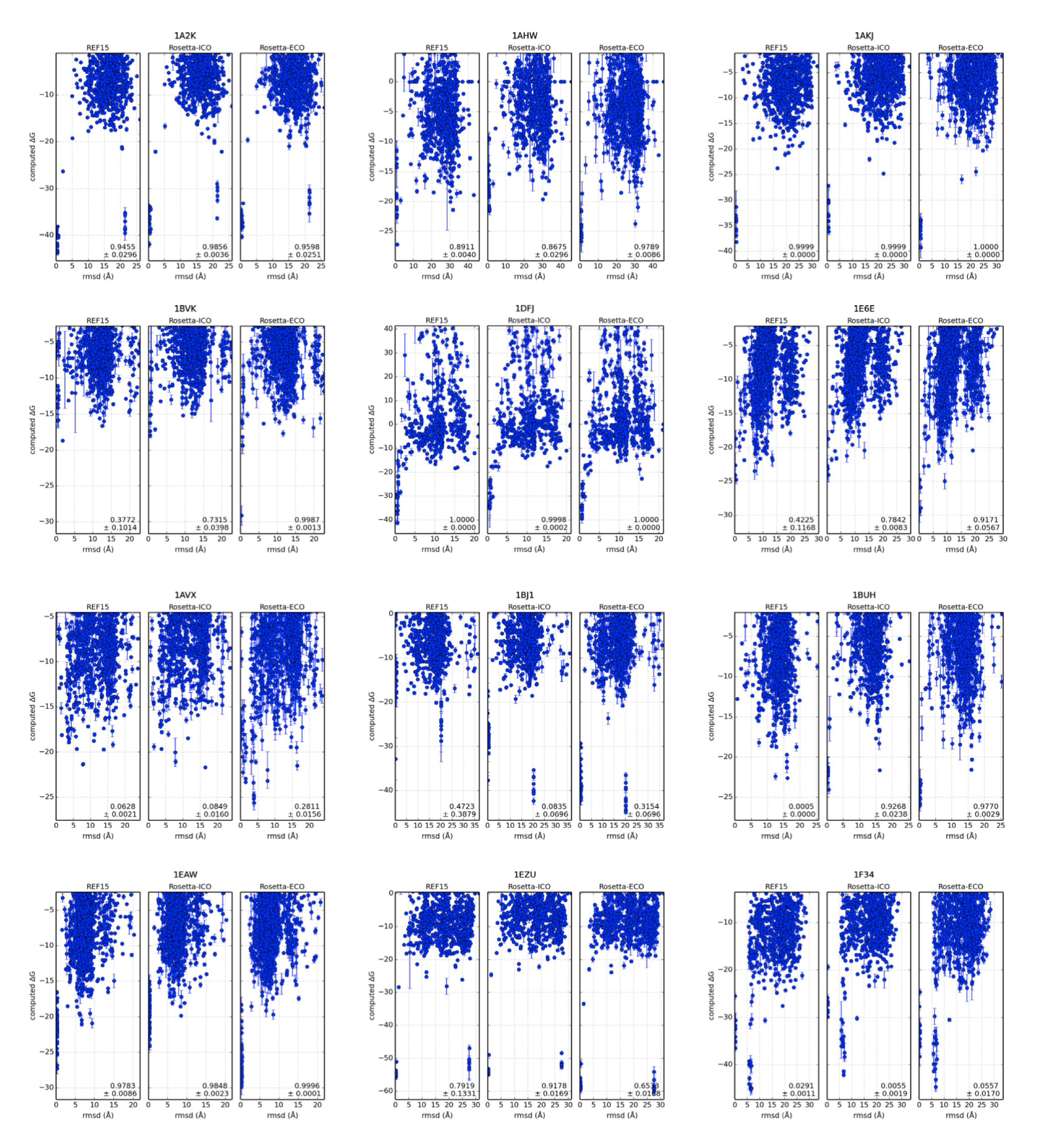

Supplement: S2 Fig — Recalculation of protein-protein docking interface scores (ΔGbind) for three different Rosetta scoring functions: REF2015, Rosetta-ICO, and Rosetta-ECO. Data points represent the average of three runs with the standard deviation as error bars. The average Boltzmann discrimination scores +/- standard deviation for each distribution is found in the bottom right corner of each plot. (TIF) [file pcbi.1008103.s003.tif]

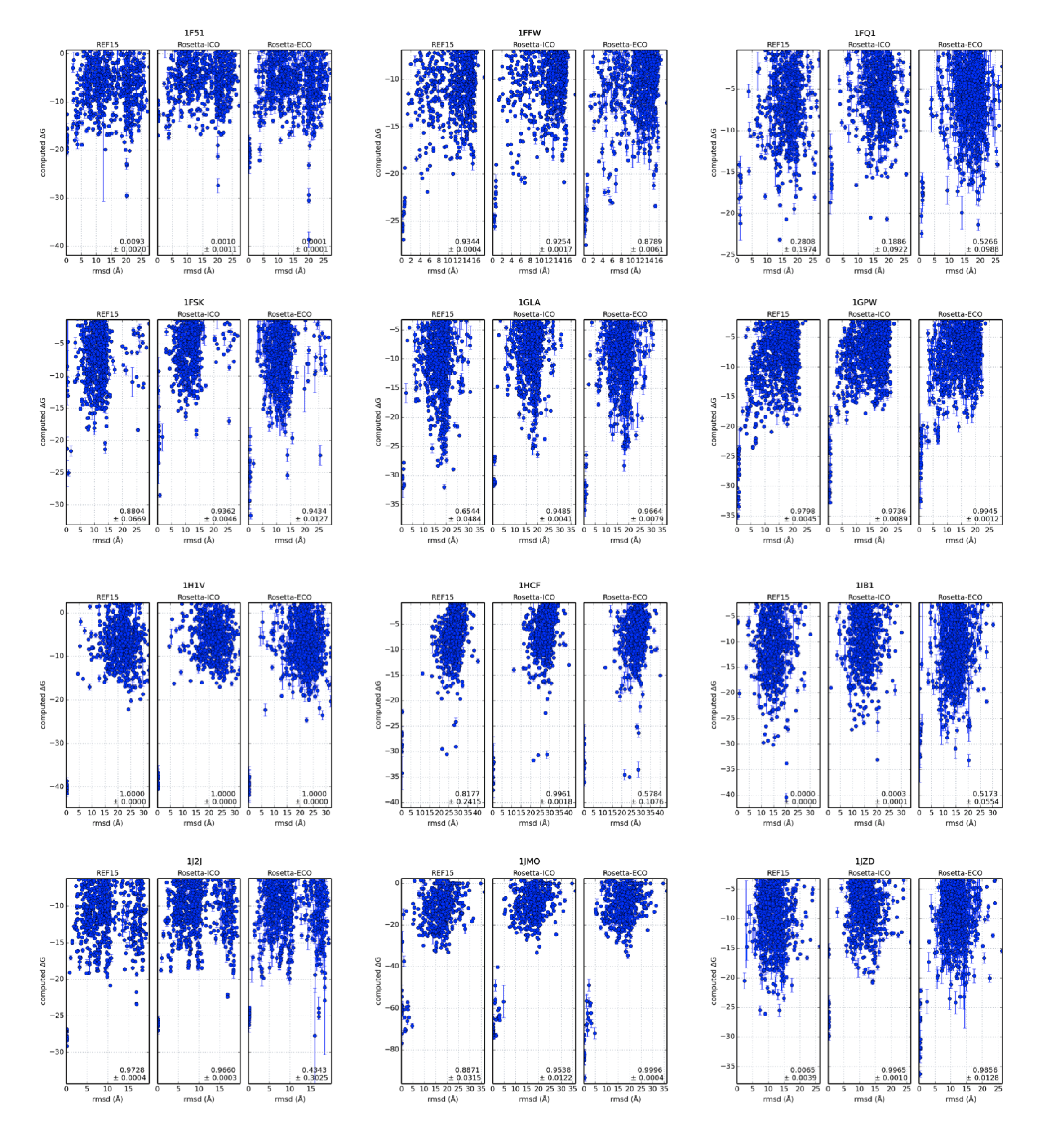

Supplement: S3 Fig — Recalculation of protein-protein docking interface scores (ΔGbind) for three different Rosetta scoring functions: REF2015, Rosetta-ICO, and Rosetta-ECO. Data points represent the average of three runs with the standard deviation as error bars. The average Boltzmann discrimination scores +/- standard deviation for each distribution is found in the bottom right corner of each plot. (TIF) [file pcbi.1008103.s004.tif]

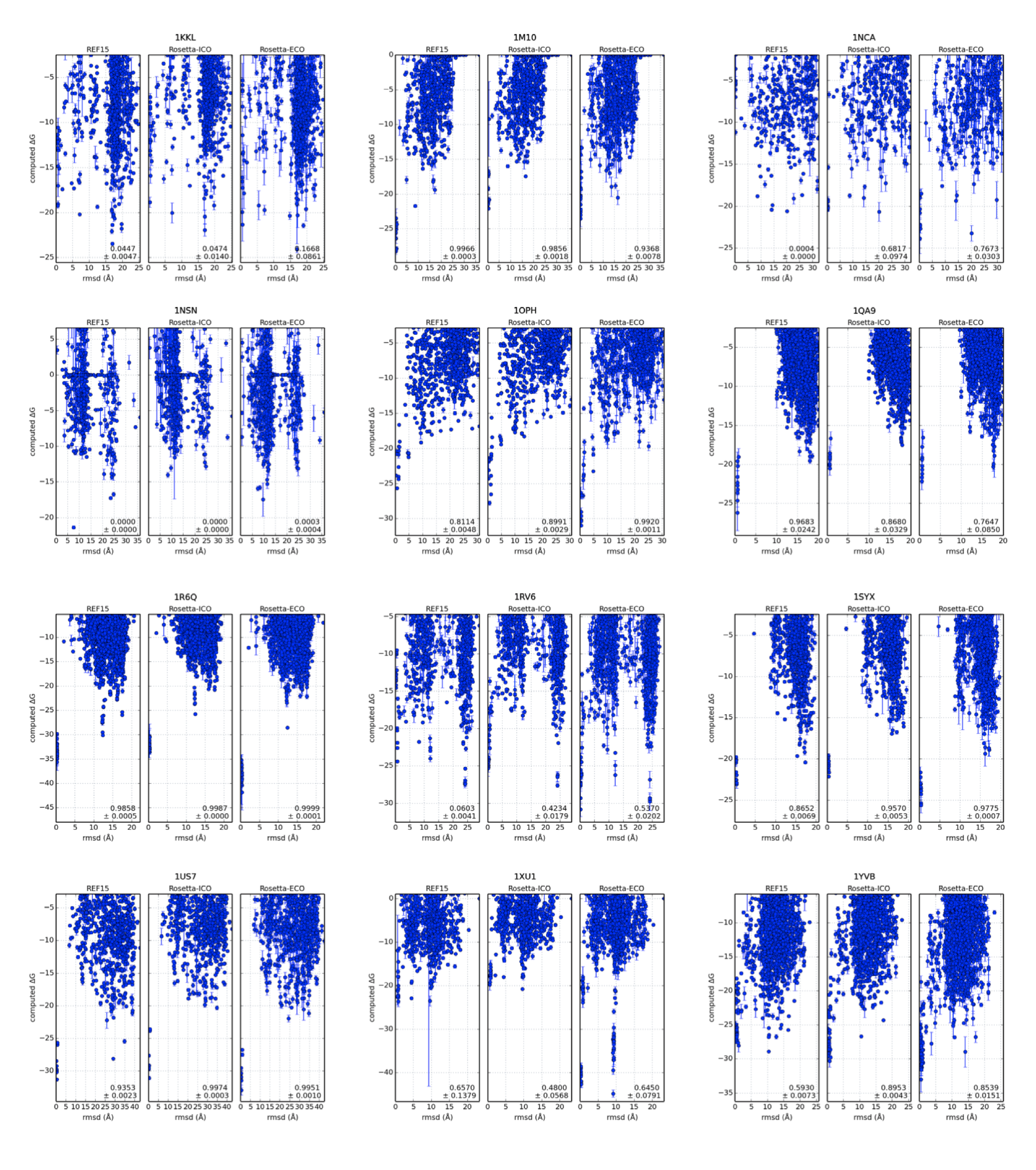

Supplement: S4 Fig — Recalculation of protein-protein docking interface scores (ΔGbind) for three different Rosetta scoring functions: REF2015, Rosetta-ICO, and Rosetta-ECO. Data points represent the average of three runs with the standard deviation as error bars. The average Boltzmann discrimination scores +/- standard deviation for each distribution is found in the bottom right corner of each plot. (TIF) [file pcbi.1008103.s005.tif]

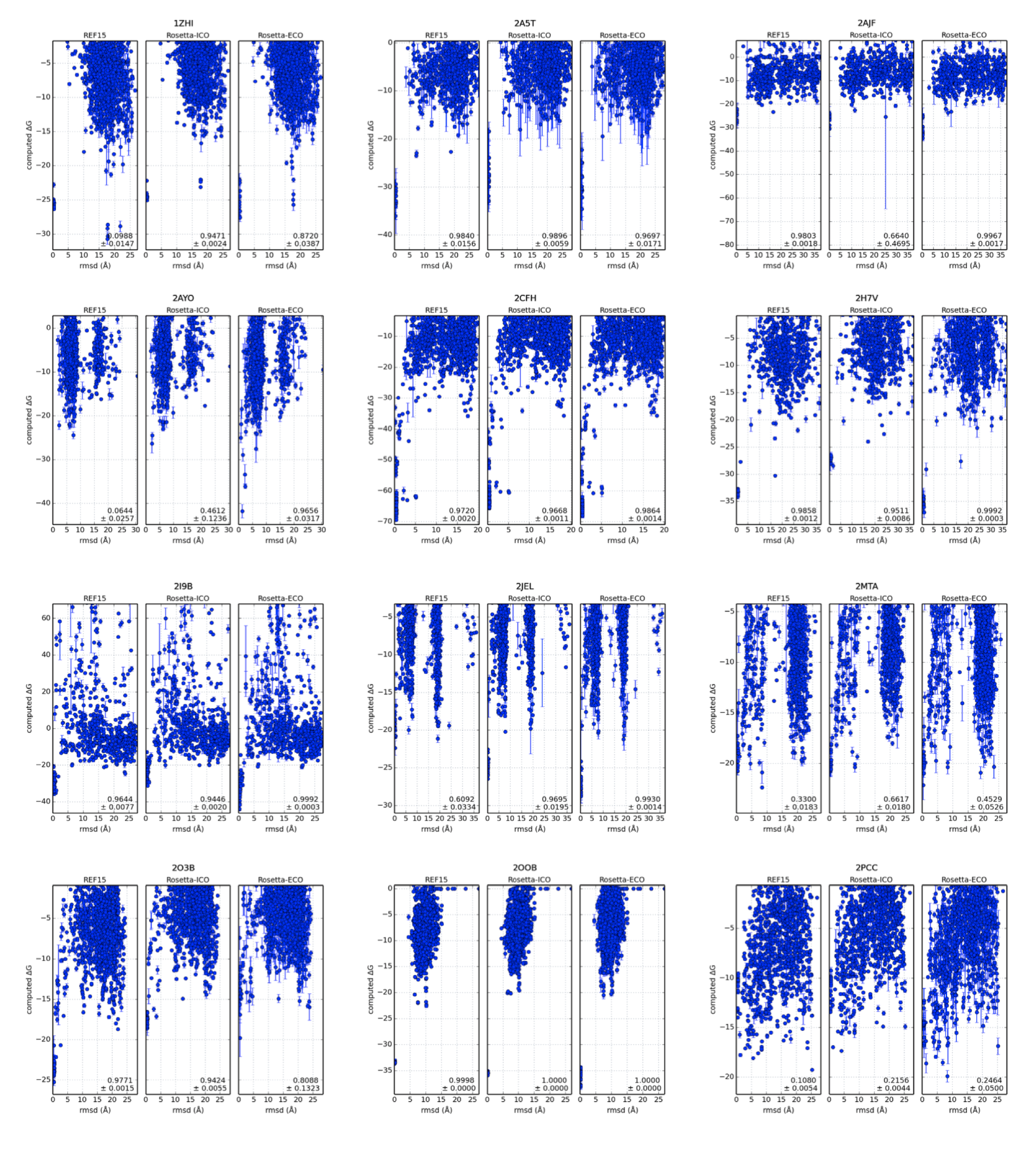

Supplement: S5 Fig — Recalculation of protein-protein docking interface scores (ΔGbind) for three different Rosetta scoring functions: REF2015, Rosetta-ICO, and Rosetta-ECO. Data points represent the average of three runs with the standard deviation as error bars. The average Boltzmann discrimination scores +/- standard deviation for each distribution is found in the bottom right corner of each plot. (TIF) [file pcbi.1008103.s006.tif]

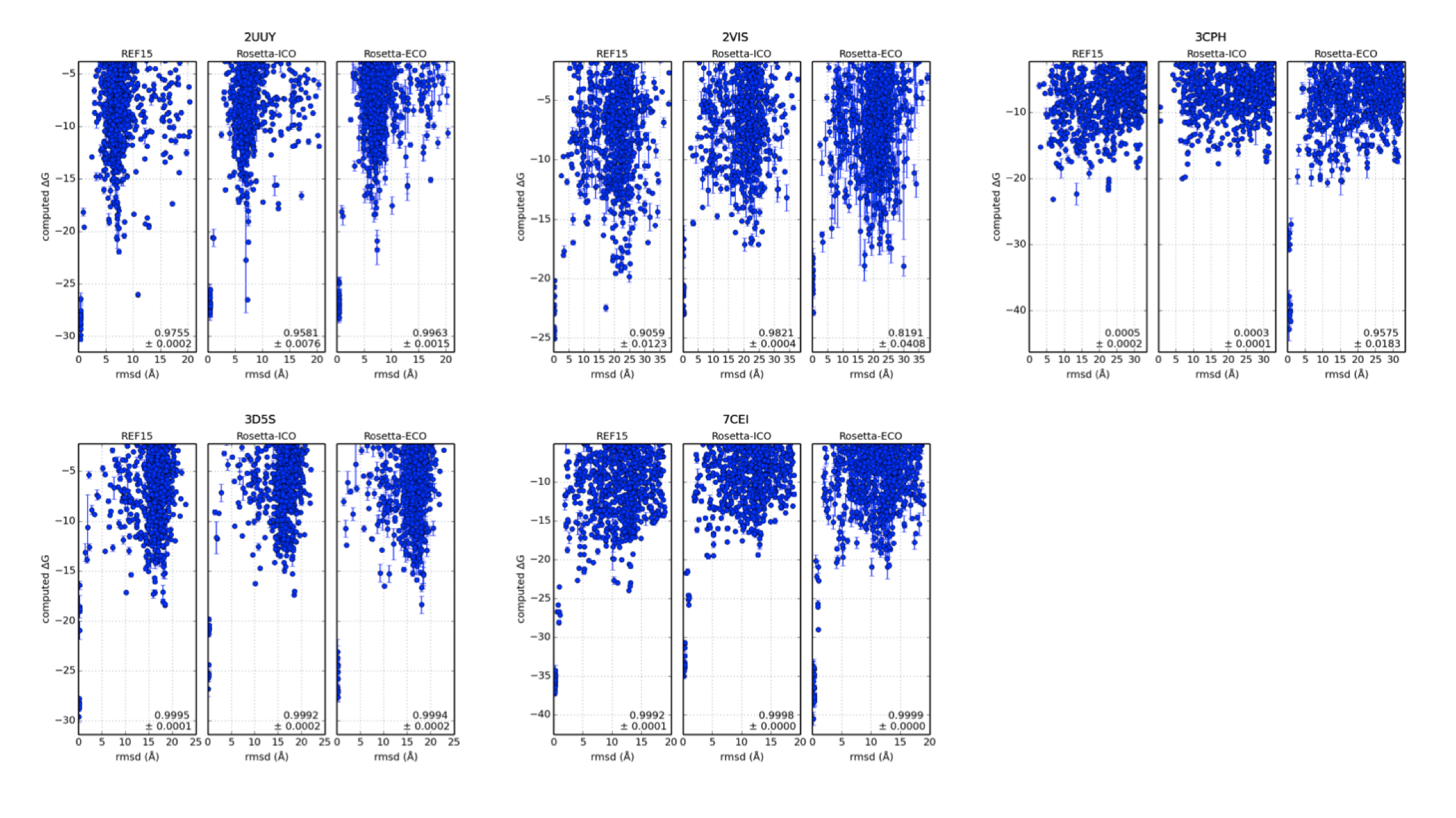

Supplement: S6 Fig — Recalculation of protein-protein docking interface scores (ΔGbind) for three different Rosetta scoring functions: REF2015, Rosetta-ICO, and Rosetta-ECO. Data points represent the average of three runs with the standard deviation as error bars. The average Boltzmann discrimination scores +/- standard deviation for each distribution is found in the bottom right corner of each plot. (TIF) [file pcbi.1008103.s007.tif]

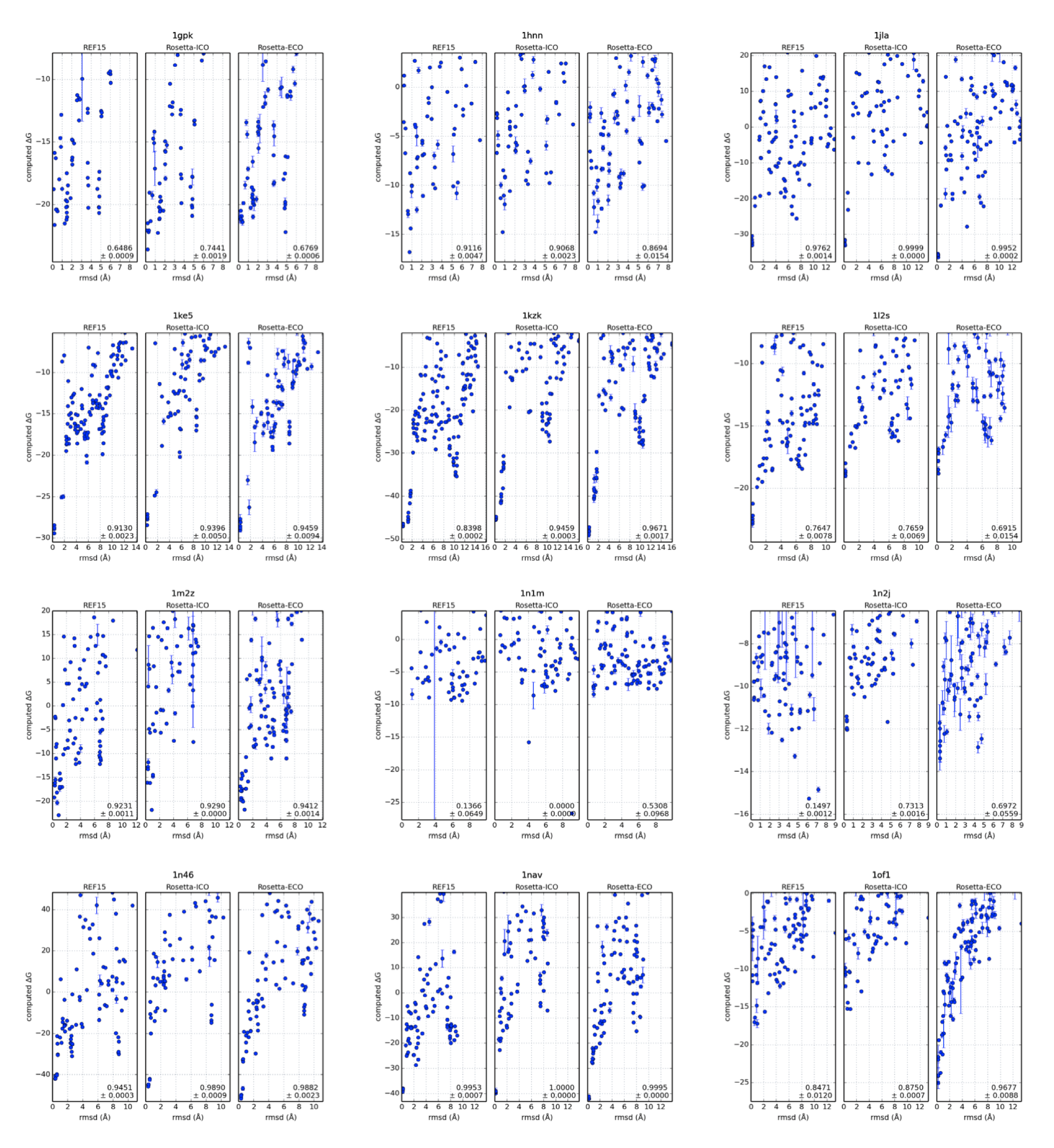

Supplement: S7 Fig — Recalculation of protein-ligand docking interface scores (ΔGbind) for three different Rosetta scoring functions: REF2015, Rosetta-ICO, and Rosetta-ECO. Data points represent the average of three runs with the standard deviation as error bars. The average Boltzmann discrimination scores +/- standard deviation for each distribution is found in the bottom right corner of each plot. (TIF) [file pcbi.1008103.s008.tif]

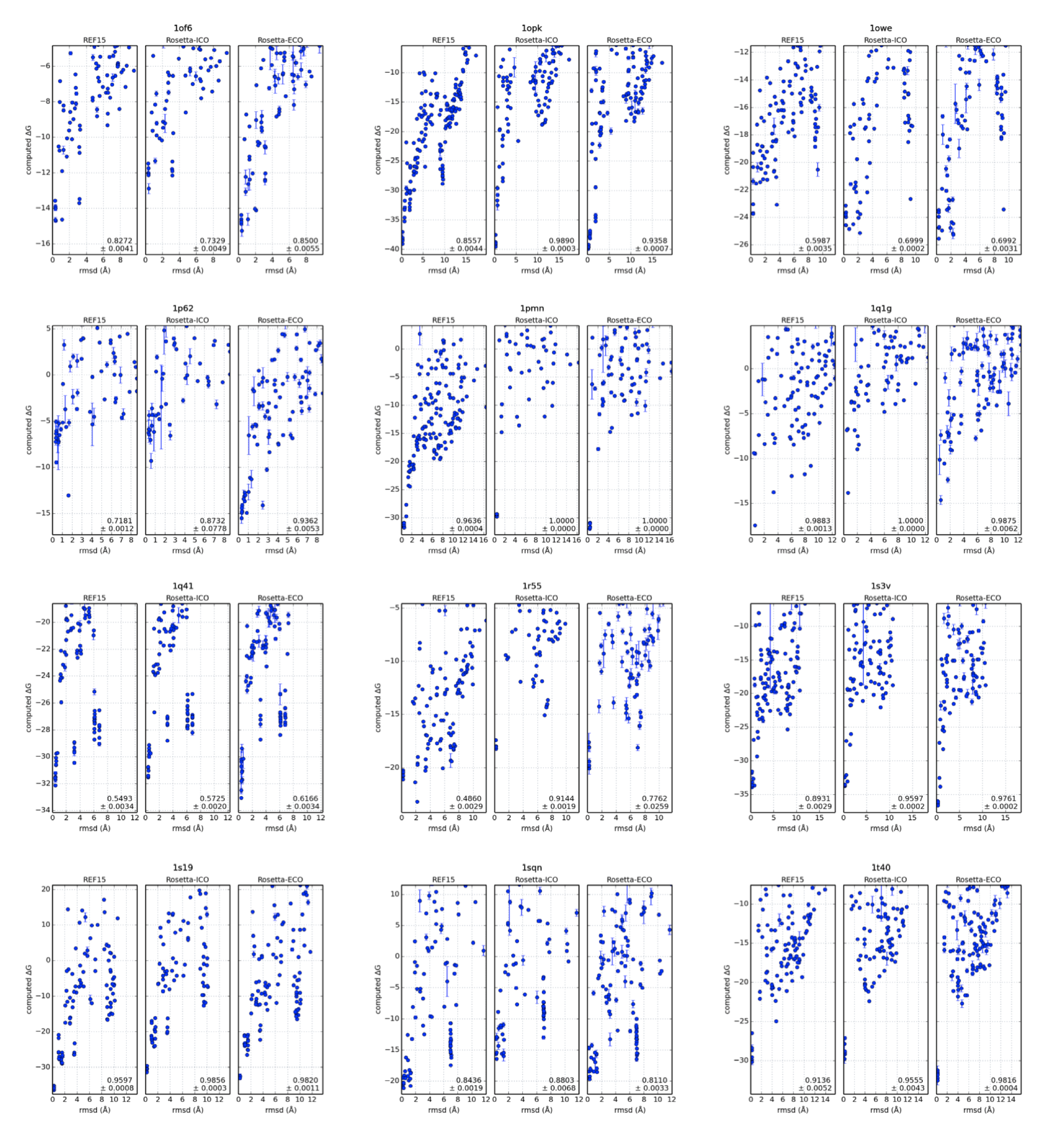

Supplement: S8 Fig — Recalculation of protein-ligand docking interface scores (ΔGbind) for three different Rosetta scoring functions: REF2015, Rosetta-ICO, and Rosetta-ECO. Data points represent the average of three runs with the standard deviation as error bars. The average Boltzmann discrimination scores +/- standard deviation for each distribution is found in the bottom right corner of each plot. (TIF) [file pcbi.1008103.s009.tif]

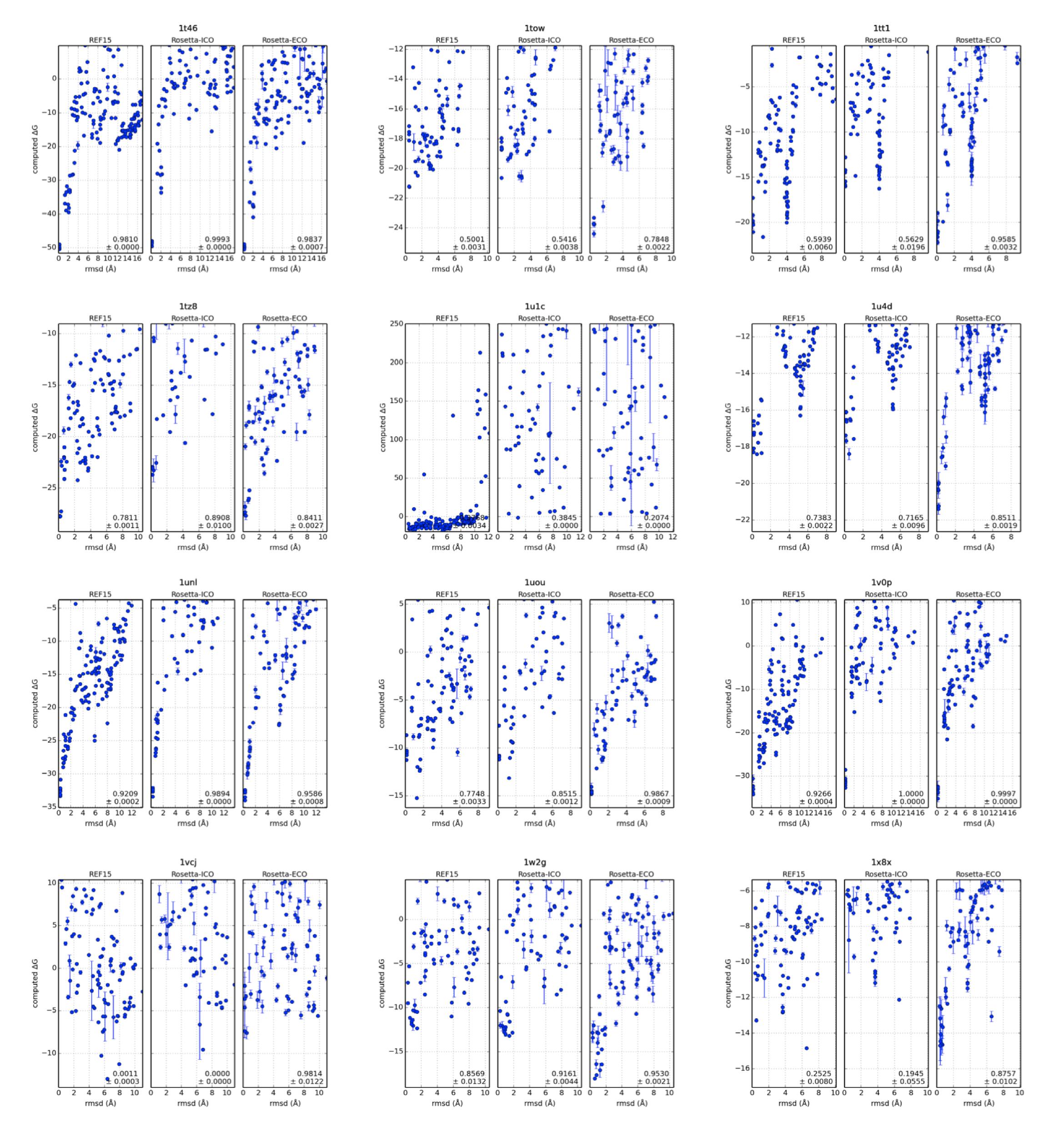

Supplement: S9 Fig — Recalculation of protein-ligand docking interface scores (ΔGbind) for three different Rosetta scoring functions: REF2015, Rosetta-ICO, and Rosetta-ECO. Data points represent the average of three runs with the standard deviation as error bars. The average Boltzmann discrimination scores +/- standard deviation for each distribution is found in the bottom right corner of each plot. (TIF) [file pcbi.1008103.s010.tif]

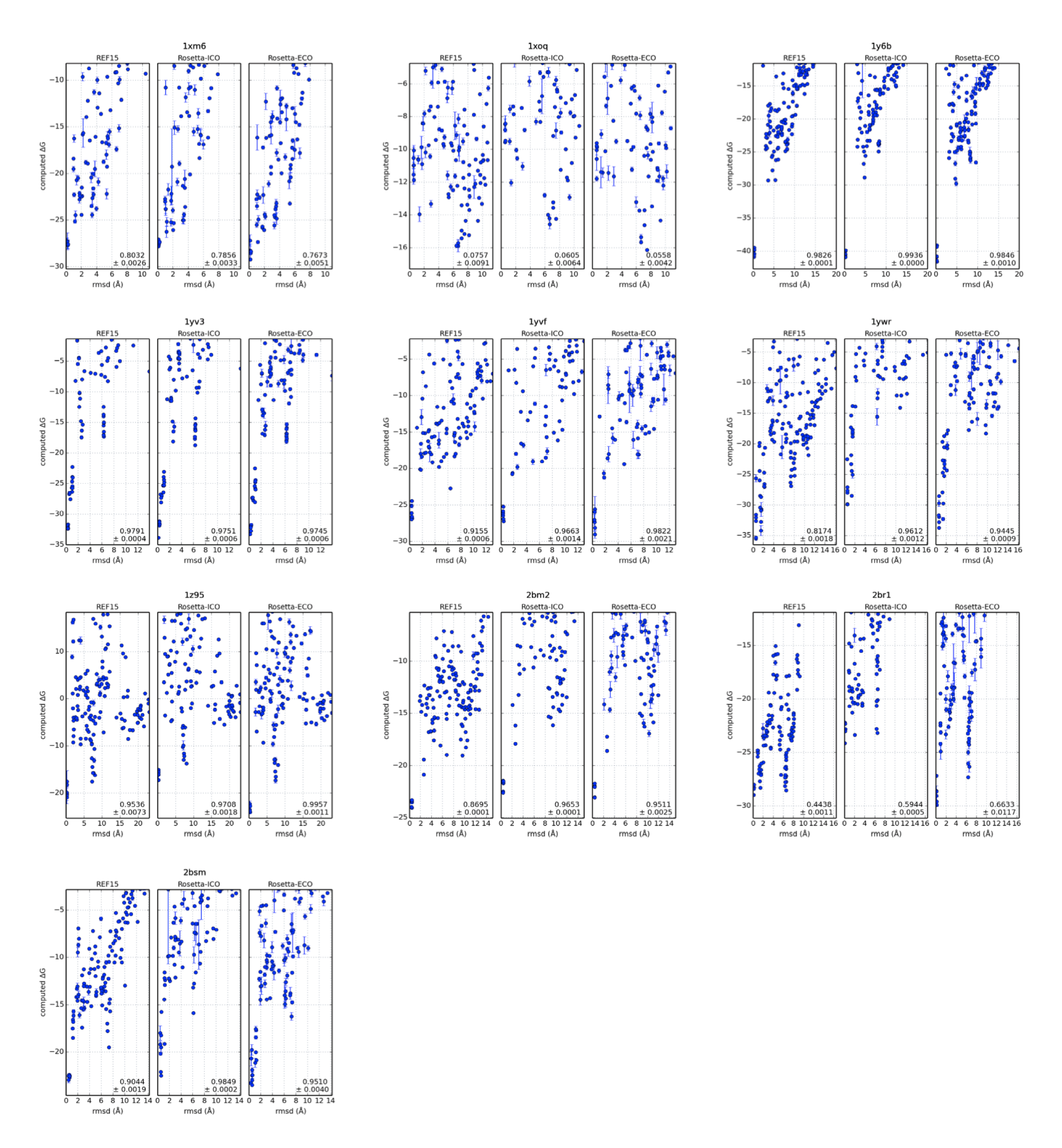

Supplement: S10 Fig — Recalculation of protein-ligand docking interface scores (ΔGbind) for three different Rosetta scoring functions: REF2015, Rosetta-ICO, and Rosetta-ECO. Data points represent the average of three runs with the standard deviation as error bars. The average Boltzmann discrimination scores +/- standard deviation for each distribution is found in the bottom right corner of each plot. (TIF) [file pcbi.1008103.s011.tif]

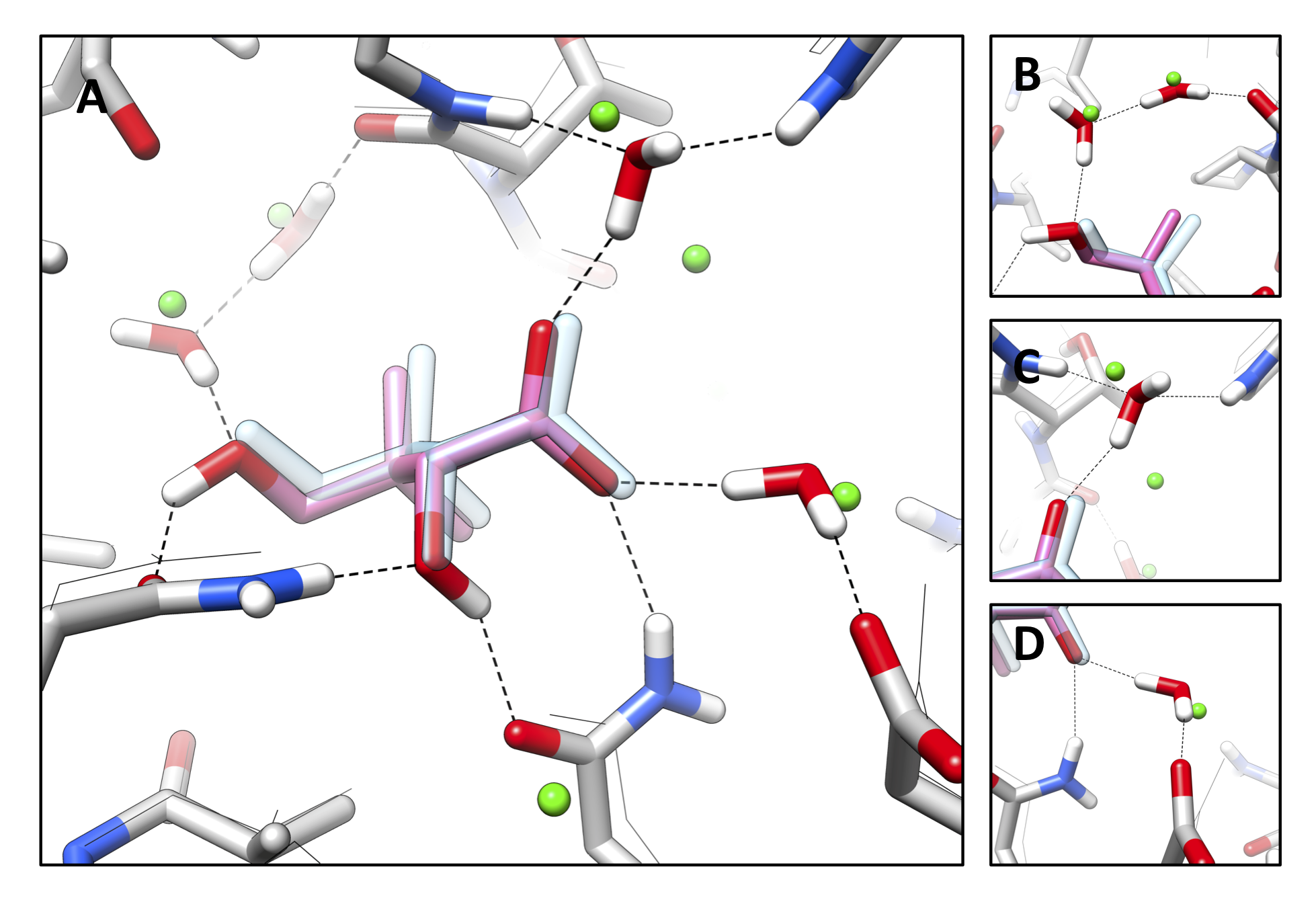

Supplement: S11 Fig — The near native Rosetta-ECO model is in thicker stick representation with full-atom water molecules and the ligand depicted in pink. The experimental ligand (pantoate) position is in transparent blue, water oxygen positions as green spheres, and native side chains are in black wire representation. If the native ligand or side chain positions cannot be seen, it is because they are obscured by the Rosetta model. Panel A highlights the overall binding pocket, while panels B-D focus on recovered water positions. (TIF) [file pcbi.1008103.s012.tif]

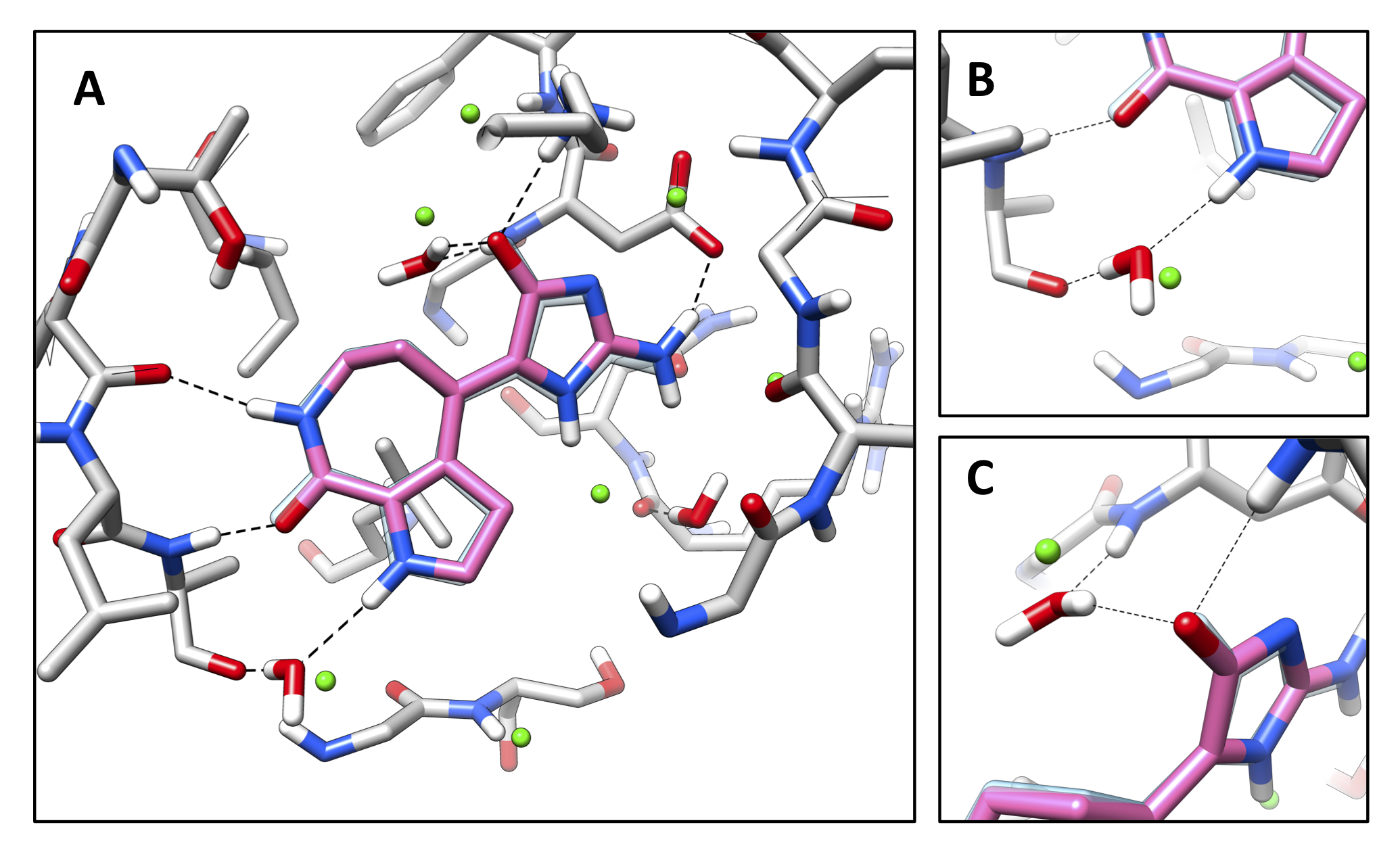

Supplement: S12 Fig — The near native Rosetta-ECO model is in thicker stick representation with full-atom water molecules and the ligand depicted in pink. The experimental ligand (debromohymenialdisine) position is in transparent blue, water oxygen positions as green spheres, and native side chains are in black wire representation. If the native ligand or side chain positions cannot be seen, it is because they are obscured by the Rosetta model. Panel A highlights the overall binding pocket, while panels B and C focus on recovered water positions. (TIF) [file pcbi.1008103.s013.tif]

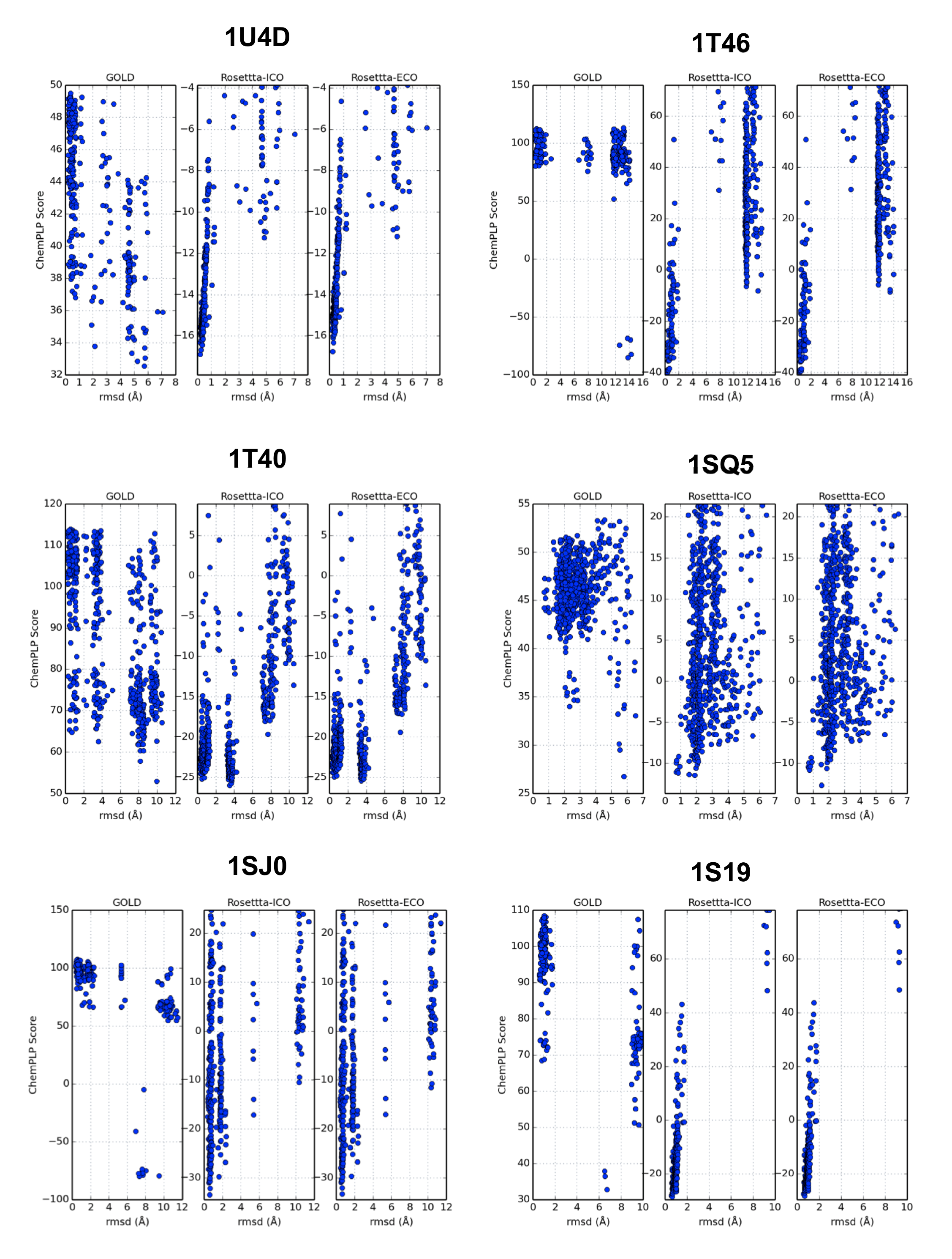

Supplement: S13 Fig — The RMSD of the ligand from the experimental conformation is plotted against the computed score (ChemPLP) for GOLD and ΔGbind for Rosetta. Note that the sampling from GOLD is often focused in small number of docking conformations, leaving gaps in the sampled space. (TIF) [file pcbi.1008103.s014.tif]

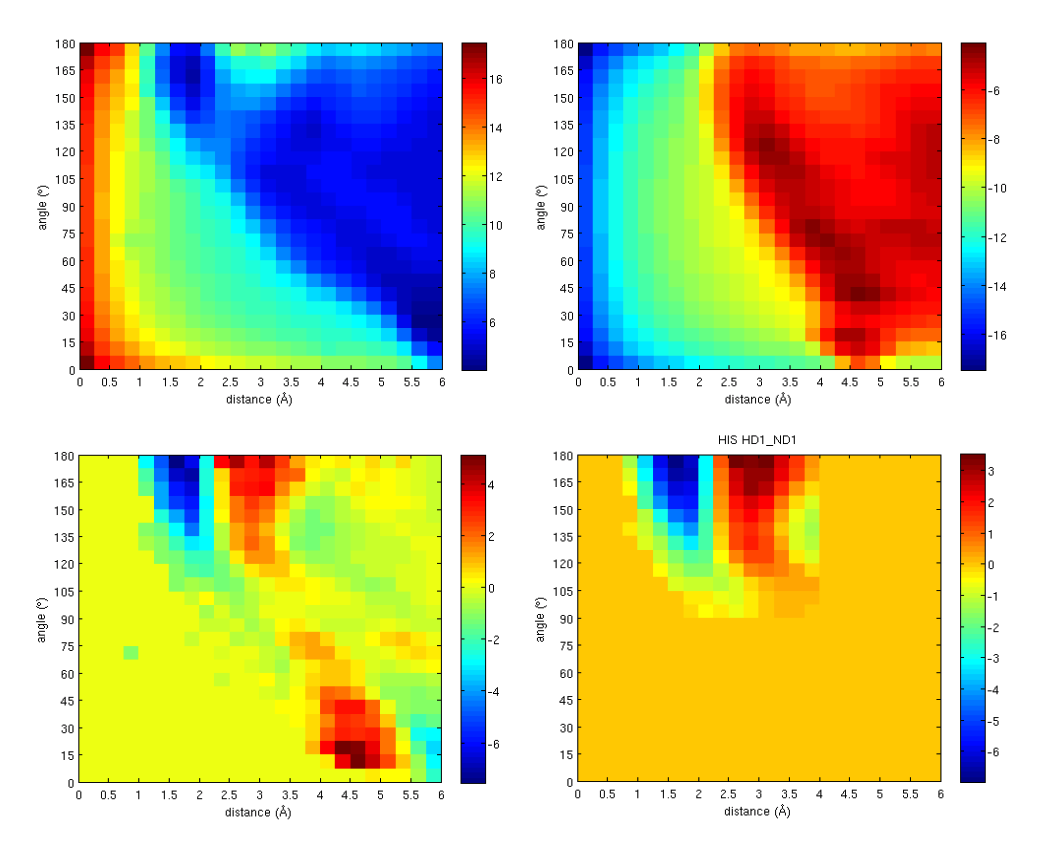

Supplement: S14 Fig — Upper left: Distribution of waters about histidine residues over a range of distance from the HD1 atom and a range of angles from the HD1 and ND1 atoms [-log(HISHD1_ND1)] Upper right: Distribution of waters about a non-polar reference [log(ALAHB1_CB1)] Lower left: The sum of the upper two figures: the statistical potential for histidine Lower right: Final, modified histidine potential filtered for noise and second solvation shell effects. (PNG) [file pcbi.1008103.s015.png]

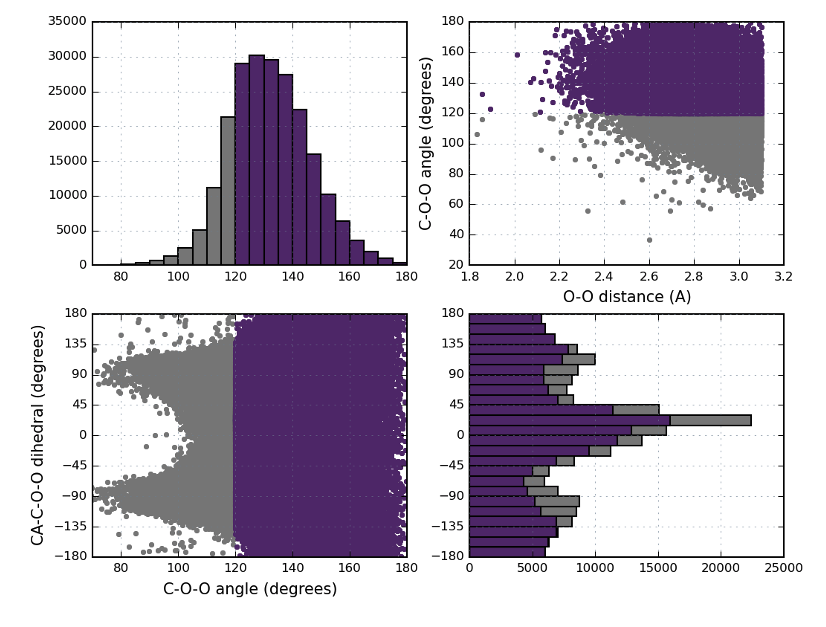

Supplement: S15 Fig — Upper right: distance and angle of all waters measured (grey) and those used for statistical placement about the polar group (purple). Bottom left: Angle and dihedral distribution with histogram projections in upper left (angle) and lower right (dihedral). (PNG) [file pcbi.1008103.s016.png]

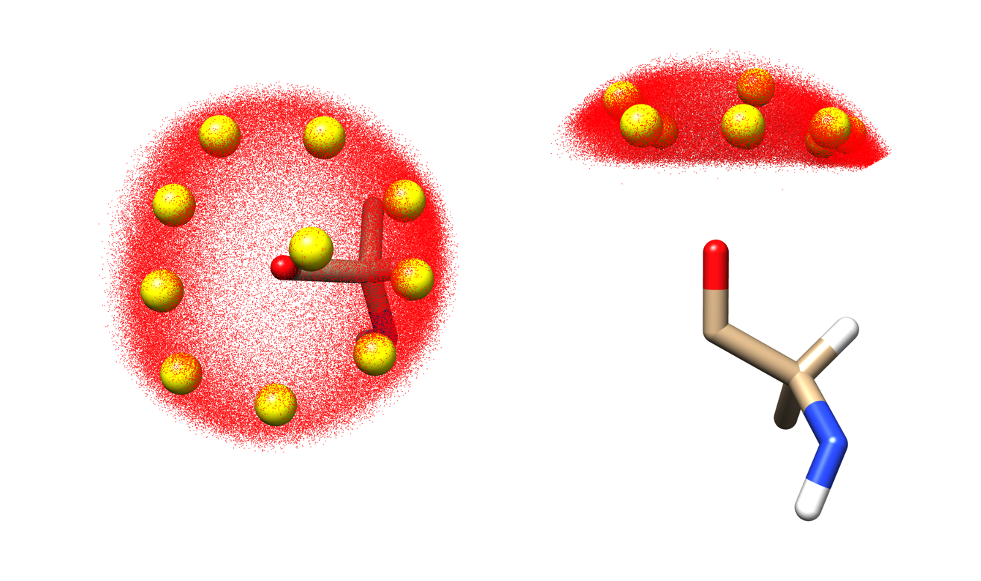

Supplement: S16 Fig — The crystallographic water positions used for statistical placement of potential solvation sites about C = O backbone polar groups are shown here in red, with the k-means cluster centroids (k = 10) illustrated in yellow. Two views of these data are shown about an arbitrary alanine residue. (PNG) [file pcbi.1008103.s017.png]

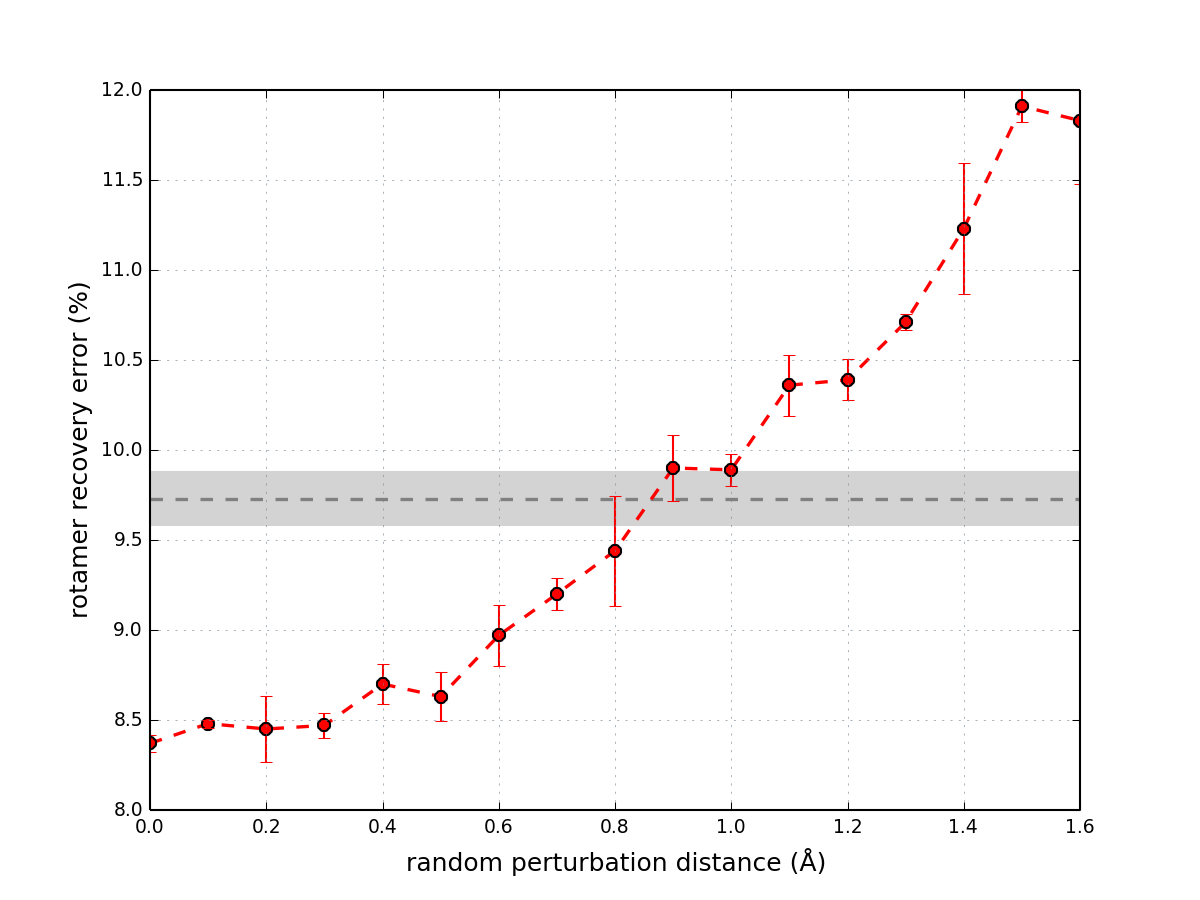

Supplement: S17 Fig — Crystallographic water molecules in our benchmark set were randomly perturbed 0.0 to 1.6 Å and the interface residues were repacked in Rosetta. Data points represent the average of three independent runs with 95% confidence interval error bars. The baseline of packing the interfaces without any water molecules (REF2015 score function) is shown as a dashed grey line with 95% confidence intervals from three runs shaded in light grey. (PNG) [file pcbi.1008103.s018.png]
