# Supplementary material for: Efficient consideration of coordinated water molecules improves computational protein-protein and protein-ligand docking discrimination
Source: PLoS Comput Biol. 2020 Sep 21;16(9):e1008103. doi: 10.1371/journal.pcbi.1008103 (PMC7529342; doi:10.1371/journal.pcbi.1008103)
Supplement: S1 Table — (DOCX) [file pcbi.1008103.s019.docx]

**S2 Table. GOLD Docking and Rosetta Rescoring Results of Astex Diverse Set**

|  | Sampling Success^1^ | | | |
| --- | --- | --- | --- | --- |
|  | 0.5 Å | 1.0 Å | 1.5 Å | 2.0 Å |
| GOLD | 79.4 ± 1.7% | 98.3 ± 0.5% | 98.4 ± 0.3% | 100 ± 0.0% |
|  | Docking Success^1^ | | | |
| GOLD | 22.4 ± 1.9% | 67.6 ± 2.5% | 75.2 ± 1.6% | 80.7 ± 1.5% |
| *REF2015* | 35.1 ± 1.6% | 78.2 ± 2.4% | 88.5 ± 1.8% | 94.6 ± 1.5% |
| *Rosetta-ICO* | 32.5 ± 1.7% | 78.2 ± 2.2% | 86.9 ± 1.4% | 93.9 ± 1.4% |
| *Rosetta-ECO* | 34.3 ± 2.0% | 77.1 ± 2.6% | 84.5 ± 1.6% | 91.2 ± 1.5% |

^1^Results are bootstrapped means ± the bootstrapped standard error of the mean from 1000 resamples of 100 individual runs, using 67 case sub-set of Astex Diverse Set.
