# Supplementary material for: Efficient consideration of coordinated water molecules improves computational protein-protein and protein-ligand docking discrimination
Source: PLoS Comput Biol. 2020 Sep 21;16(9):e1008103. doi: 10.1371/journal.pcbi.1008103 (PMC7529342; doi:10.1371/journal.pcbi.1008103)
Supplement: S2 Table — (DOCX) [file pcbi.1008103.s020.docx]

**Table S3. 3D-RISM Results on Interface Water Test Set**

|  |  | 3D-RISM^1^ | |
| --- | --- | --- | --- |
| Type^2^ | Subset Size | % recovered^3^ | % precision^4^ |
| All | 3226 | 22.9 | 17.8 |
| Exposed | 773 | 10.5 | 12.1 |
| Partially Buried | 2046 | 25.0 | 20.4 |
| Buried | 407 | 36.4 | 15.0 |
| 1 protein coord | 892 | 2.5 | 4.0 |
| 2 protein coord | 1219 | 30.7 | 27.4 |
| 3 protein coord | 458 | 53.9 | 18.9 |
| BB only | 818 | 14.7 | 10.6 |
| SC only | 814 | 19.8 | 18.4 |
| BB+SC | 1070 | 42.8 | 23.3 |

^1^3-D RISM water positions with placevent algorithm[35] using RDF cutoff of 10.2 and trimmed within 6 Å of CB atoms (CA for GLY) of interface residues

^2^Three groups of categorization of type of predicted water molecules. First, waters are classified ‘buriedness’ based on number of amino acid neighbors (nCβ) with Cβ within 10 Å. Exposed: nCβ <=15; partially buried: 15 < nCβ <= 25; buried: nCβ > 25. Second, classification by 1, 2, or 3 protein coordination partners within 3.2 Å. Finally, by type of coordinating protein atoms with 3.2 Å of the water O atom: at least two backbone only (BB only), side chain only (SC only) or a mix of backbone and side chain coordination (BB+SC).

^3-4^Percent and number of specific types of waters recovered using recovery criteria (0.5 Å) as described in *Methods*, averaged over three runs.
