# Supplementary material for: Efficient consideration of coordinated water molecules improves computational protein-protein and protein-ligand docking discrimination
Source: PLoS Comput Biol. 2020 Sep 21;16(9):e1008103. doi: 10.1371/journal.pcbi.1008103 (PMC7529342; doi:10.1371/journal.pcbi.1008103)
Supplement: S3 Table — (DOCX) [file pcbi.1008103.s021.docx]

**S4 Table. Timing Comparison Between *Rosetta-ECO* and 3D-RISM**

| PDB ID | Interface size (Å^2^) | # of interface residues | Ave. baseline Rosetta (REF2015) time (s) | Ave. *Rosetta-ECO* time (s) | Fold change of runtime between REF15 and ECO | Ave 3D-RISM time (s) | Fold change of runtime between *ECO* and 3D-RISM |
| --- | --- | --- | --- | --- | --- | --- | --- |
| 4DRI | 772.0 | 10 | 11.6 (1.2) | 16.2 (1.0) | 1.4 | 369.6 (10.0) | 22.8 |
| 1PK1 | 968.0 | 19 | 13.0 (0.6) | 20.0 (2.4) | 1.5 | 174.7 (1.9) | 8.7 |
| 3BC1 | 2017.2 | 30 | 14.8 (1.5) | 23.2 (2.6) | 1.6 | 391.9 (4.1) | 16.9 |
| 1MCT | 1526.2 | 40 | 12.0 (0.0) | 18.6 (1.2) | 1.6 | 338.8 (5.2) | 18.2 |
| 1P57 | 2477.2 | 50 | 20.8 (1.8) | 42.8 (3.9) | 2.1 | 655.5 (6.0) | 15.3 |
| 3MMY | 3004.8 | 60 | 25.6 (0.8) | 51.0 (3.7) | 2.0 | 792.5 (7.8) | 15.5 |
| 1G8K | 3943.3 | 75 | 22.2 (3.5) | 51.0 (12.7) | 2.3 | 2994.1 (117.3) | 58.7 |
| 2NW2 | 4504.9 | 105 | 26.6 (2.5) | 59.8 (10.0) | 2.2 | 1061.9 (28.8) | 17.8 |
| 1E3D | 7217.4 | 154 | 35.8 (2.2) | 89.4 (17.3) | 2.5 | 2326.5 (50.0) | 26.0 |
| average | | | | | 1.9 (0.4) |  | 22.2 (13.6) |

All calculations performed on a single processor of the same machine. Average times, in seconds, are based off of three independent runs with standard deviations in parentheses.
