# Supplementary material for: Efficient consideration of coordinated water molecules improves computational protein-protein and protein-ligand docking discrimination
Source: PLoS Comput Biol. 2020 Sep 21;16(9):e1008103. doi: 10.1371/journal.pcbi.1008103 (PMC7529342; doi:10.1371/journal.pcbi.1008103)
Supplement: S1 Text — Final parameters used in Rosetta-ICO and ECO force fields. (DOCX) [file pcbi.1008103.s022.docx]

**S1 Rosetta Force Field Parameters**

**Final *Rosetta-ICO* force field parameters:**

**Table S7. Lennard Jones radius parameters for fa_atr and fa_rep terms**

| **Atom Type** | **REF2015 LJ_RADIUS (kcal/mol)** | ***Rosetta-ICO* LJ_RADIUS (kcal/mol)** |
| --- | --- | --- |
| aroC | 2.0164 | 1.9991 |
| CAbb | 2.0118 | 2.0218 |
| CH0 | 2.0118 | 2.0218 |
| CH1 | 2.0118 | 2.0218 |
| CH2 | 2.0118 | 2.0711 |
| CH3 | 2.0118 | 2.0235 |
| CNH2 | 1.9683 | 2.0158 |
| CObb | 1.9167 | 1.9251 |
| COO | 1.9167 | 1.9251 |
| Narg | 1.8025 | 1.7534 |
| Nbb | 1.8025 | 1.7535 |
| NH20 | 1.8025 | 1.7535 |
| Nhis | 1.8025 | 1.7535 |
| Nlys | 1.8025 | 1.7535 |
| Npro | 1.8025 | 1.7535 |
| Ntrp | 1.8025 | 1.7535 |
| NtrR | 1.8025 | 1.7535 |
| OCbb | 1.5406 | 1.5505 |
| OH | 1.5427 | 1.5505 |
| OW | 1.5427 | 1.5505 |
| ONH2 | 1.5487 | 1.5505 |
| OOC | 1.4929 | 1.4938 |
| S | 1.9760 | 1.8825 |
| SH1 | 1.9760 | 1.8825 |

**Table S8. Lennard Jones well-depth parameters for fa_atr and fa_rep terms**

| **Atom Type** | **REF2015 ΔG^free^ (kcal/mol)** | ***Rosetta-ICO* ΔG^free^ (kcal/mol)** |
| --- | --- | --- |
| aroC | 0.0688 | 0.1194 |
| CAbb | 0.0626 | 0.0553 |
| CH0 | 0.0626 | 0.0553 |
| CH1 | 0.0626 | 0.0553 |
| CH2 | 0.0626 | 0.1438 |
| CH3 | 0.0626 | 0.2409 |
| CNH2 | 0.0946 | 0.0802 |
| CObb | 0.1418 | 0.1173 |
| COO | 0.1418 | 0.1173 |
| Narg | 0.1617 | 0.1701 |
| Nbb | 0.1617 | 0.1701 |
| NH20 | 0.1617 | 0.1701 |
| Nhis | 0.1617 | 0.1701 |
| Nlys | 0.1617 | 0.1701 |
| Npro | 0.1617 | 0.1701 |
| Ntrp | 0.1617 | 0.1701 |
| NtrR | 0.1617 | 0.1701 |
| OCbb | 0.1424 | 0.1611 |
| OH | 0.1619 | 0.1601 |
| OW | 0.1619 | 0.1601 |
| ONH2 | 0.1829 | 0.1611 |
| OOC | 0.0998 | 0.2132 |
| S | 0.4560 | 0.4332 |
| SH1 | 0.4560 | 0.4332 |

**Table S9. ΔG^free^ parameters for Lazaridis-Karplus solvation term**

| **Atom Type** | **REF2015 ΔG^free^ (kcal/mol)** | ***Rosetta-ICO* ΔG^free^ (kcal/mol)** |
| --- | --- | --- |
| aroC | 1.7979 | 2.2228 |
| CAbb | 2.5338 | 4.4495 |
| CH0 | 1.4093 | 1.2487 |
| CH1 | -3.5384 | -6.4922 |
| CH2 | -1.8547 | -2.5518 |
| CH3 | 7.2929 | 7.7272 |
| CNH2 | 3.0770 | 3.7033 |
| CObb | 3.1042 | 3.5790 |
| COO | -3.3326 | -2.5088 |
| Narg | -8.9684 | -8.6960 |
| Nbb | -9.9695 | -12.8467 |
| NH20 | -8.1016 | -7.6667 |
| Nhis | -9.7396 | -9.7253 |
| Nlys | -20.865 | -18.7433 |
| Npro | -0.9846 | -1.5111 |
| Ntrp | -8.4131 | -10.6448 |
| NtrR | -5.1581 | -4.9280 |
| OCbb | -8.0068 | -9.5292 |
| OH | -8.1335 | -5.4606 |
| OW | -8.1335 | -5.4606 |
| ONH2 | -6.5916 | -5.0350 |
| OOC | -9.2398 | -10.2082 |
| S | -1.7072 | -4.8980 |
| SH1 | 3.2916 | 2.0795 |

**Table S10. Scale factors for partial atomic charges of amino acid groups with respect to the charge sets used in the REF2015 score function.**

| **Atom Group** | **Partial Charge Scale Factor^1^** |
| --- | --- |
| THR side chain | 0.93098831 |
| TYR side chain | 0.81613842 |
| ASP side chain | 1.16679674 |
| HIS side chain | 0.94011469 |
| GLU side chain | 0.84384323 |
| ASN side chain | 0.81255557 |
| GLN side chain | 1.07403123 |
| SER side chain | 0.81598146 |
| PHE side chain | 1.59168337 |
| TRP side chain | 1.20396806 |
| LYS side chain | 0.97974623 |
| ARG side chain | 0.59867274 |
| nonpolar side chains | 1.35107606 |
| all CO groups | 1.49998495 |
| all NH groups | 1.63951702 |
| nterm NH | 0.58134422 |
| cterm CO | 1.02204412 |

^1^Net charge on side chains remains preserved. The scale factors greater than 1 concentrate charge, while scale factors less than 1 distribute the charge with resepct to the REF2015 charge sets.
