# Supplementary material for: Efficient consideration of coordinated water molecules improves computational protein-protein and protein-ligand docking discrimination
Source: PLoS Comput Biol. 2020 Sep 21;16(9):e1008103. doi: 10.1371/journal.pcbi.1008103 (PMC7529342; doi:10.1371/journal.pcbi.1008103)
Supplement: S2 Text — Details on datasets used for water recovery and docking discrimination tests, including GOLD docking protocol. (DOCX) [file pcbi.1008103.s023.docx]

**Dataset Details:**

**1.) Water and Interface Rotamer Recovery Datasets**

A set of 153 high-resolution X-ray crystal structures (resolution <= 1.8 Å) of protein-protein complexes were used for water prediction and interface rotamer recovery testing. The complexes are all heteromeric, excluding all antibody structures which were over-represented in initial dataset building. Interface waters were filtered such that each water comes within 3.5 Å of a heavy atom of both chains forming the interface or forms a three-water bridge between the two chains with the anchoring waters coming within 3.5 Å of each chain. An additional filter was used to remove water molecules that clash with one another. Waters within 0.85*2*O_vdw_ of each other, where O_vdw_ is the van der Waals radius of the water oxygen atom (1.55 Å), were checked for how well they fit into the electron density map of the crystal structure. Any clashing water with an electron density correlation < 3.5 was removed from the final set of interface waters.

Interface residues were defined by the RestrictToInterfaceVector task operation in Rosetta, using the following parameters: vector_dist_cutoff = 9.0; vector_angle_cutoff = 75.0; nearby_atom_cutoff = 5.5; CB_dist_cutoff = 10.0. These residues were solvated with the two-stage method described above. Following the final repack with the full-atom energy function in Rosetta on a fixed backbone, the final computationally determined side-chain conformations were compared to the experimental structure to measure recovery of rotameric states. Only residues with experimental side-chain conformations that correlate well to the electron density map (correlation score ≥ 0.72) were used for analysis. A total of 7040 side chain rotamers from the test set met this criterion. Predicted side-chain conformations were determined to the native conformation if the difference in electron density correlation was less than or equal to 0.12 with an individual density correlation greater than or equal to 0.71.

Predicted water positions were determined to recall one of the 3290 native interface water molecules if the oxygen position was within 0.5 Å of crystallographic position or if the predicted water coordinates that same neighboring polar groups as a native using a 3.5 Å heavy atom cutoff. As indicated in Table S1, a subset of 30 structures was used for parameter training, while the remaining 123 structures were used for validation (Table S1 in this document).

**Table S1. Native Interface Water Dataset Details**

|  | PDB | # Native Waters^1^ | # Predicted Waters^2^ | Interface Size (Å^2^)^3^ | # water / 1000 Å^2^ | Year | Resolution (Å) |
| --- | --- | --- | --- | --- | --- | --- | --- |
| 1 | 3FJU | 49 | 14 | 1399.2 | 35.0 | 2008 | 1.6 |
| 2 | 3G9A | 44 | 16 | 1348.3 | 32.6 | 2009 | 1.614 |
| 3 | 4CBU | 51 | 14 | 2049.1 | 24.9 | 2013 | 1.3 |
| 4 | 2WBW | 35 | 26 | 1426.3 | 24.5 | 2009 | 1.55 |
| 5 | 1T6G | 45 | 32 | 1986.6 | 22.7 | 2004 | 1.8 |
| 6 | 4KT3 | 57 | 24 | 2658.8 | 21.4 | 2013 | 1.4362 |
| 7 | 4K12 | 27 | 13 | 1345.1 | 20.1 | 2013 | 1.49 |
| 8 | 4EQA | 36 | 23 | 1815.5 | 19.8 | 2012 | 1.6 |
| 9 | 4K5A | 32 | 9 | 1712.1 | 18.7 | 2013 | 1.5 |
| 10 | 1WMH | 23 | 7 | 1239.0 | 18.6 | 2004 | 1.5 |
| 11 | 1F60 | 64 | 35 | 3534.4 | 18.1 | 2000 | 1.67 |
| 12 | 3N1F | 23 | 15 | 1326.6 | 17.3 | 2010 | 1.6 |
| 13 | 4APX | 34 | 25 | 1963.9 | 17.3 | 2012 | 1.65 |
| 14 | 2Y5F | 30 | 18 | 1749.3 | 17.1 | 2011 | 1.29 |
| 15 | 1JIW | 36 | 25 | 2104.6 | 17.1 | 2001 | 1.74 |
| 16 | 2OMZ | 50 | 30 | 2934.0 | 17.0 | 2007 | 1.6 |
| 17 | 2VSM | 45 | 30 | 2846.5 | 15.8 | 2008 | 1.8 |
| 18 | 2V9T | 23 | 17 | 1463.2 | 15.7 | 2007 | 1.7 |
| 19 | 2VLQ | 23 | 16 | 1508.2 | 15.2 | 2008 | 1.6 |
| 20 | 2HQS | 35 | 27 | 2356.4 | 14.9 | 2006 | 1.5 |
| 21 | 2FHZ | 35 | 20 | 2453.0 | 14.3 | 2005 | 1.15 |
| 22 | 1L6X | 20 | 11 | 1426.6 | 14.0 | 2002 | 1.65 |
| 23 | 3D3B | 24 | 15 | 1722.0 | 13.9 | 2008 | 1.3 |
| 24 | 2UYZ | 17 | 15 | 1249.7 | 13.6 | 2007 | 1.4 |
| 25 | 4HI8 | 26 | 8 | 1914.4 | 13.6 | 2012 | 1.203 |
| 26 | 2IEJ | 92 | 54 | 6797.3 | 13.5 | 2006 | 1.8 |
| 27 | 1R0R | 19 | 18 | 1405.3 | 13.5 | 2003 | 1.1 |
| 28 | 2BCG | 39 | 31 | 2885.3 | 13.5 | 2005 | 1.48 |
| 29 | 4HT3 | 36 | 32 | 2724.4 | 13.2 | 2012 | 1.3 |
| 30 | 4GVB | 12 | 7 | 910.7 | 13.2 | 2012 | 1.8 |
| 31 | 3KYJ | 14 | 12 | 1066.0 | 13.1 | 2009 | 1.4 |
| 32 | 2QWO | 13 | 12 | 994.1 | 13.1 | 2007 | 1.7 |
| 33 | 2ZA4 | 19 | 18 | 1464.4 | 13.0 | 2007 | 1.58 |
| 34 | 4KVG | 10 | 15 | 773.7 | 12.9 | 2013 | 1.65 |
| 35 | 3L51 | 19 | 15 | 1471.9 | 12.9 | 2009 | 1.506 |
| 36 | 3LXR | 35 | 27 | 2723.8 | 12.8 | 2010 | 1.68 |
| 37 | 2H7Z | 11 | 12 | 875.9 | 12.6 | 2006 | 1.5 |
| 38 | 3P95 | 16 | 15 | 1309.0 | 12.2 | 2010 | 1.2991 |
| 39 | 3C9A | 32 | 25 | 2669.5 | 12.0 | 2008 | 1.6 |
| 40 | 4JZZ | 12 | 21 | 1002.2 | 12.0 | 2013 | 1.49 |
| 41 | 1H32 | 31 | 18 | 2591.7 | 12.0 | 2002 | 1.5 |
| 42 | 3EGV | 35 | 21 | 2934.9 | 11.9 | 2008 | 1.75 |
| 43 | 1LQV | 11 | 12 | 925.5 | 11.9 | 2002 | 1.6 |
| 44 | 3N4I | 31 | 27 | 2669.0 | 11.6 | 2010 | 1.56 |
| 45 | 3AWU | 27 | 18 | 2336.2 | 11.6 | 2011 | 1.16 |
| 46 | 4KT6 | 39 | 32 | 3384.5 | 11.5 | 2013 | 1.71 |
| 47 | 4LGR | 16 | 10 | 1389.0 | 11.5 | 2013 | 1.65 |
| 48 | 3NHE | 46 | 35 | 4058.2 | 11.3 | 2010 | 1.26 |
| 49 | 4L2I | 85 | 59 | 7514.6 | 11.3 | 2013 | 1.45 |
| 50 | 4NBX | 16 | 18 | 1430.5 | 11.2 | 2013 | 1.75 |
| 51 | 2Z7F | 17 | 20 | 1520.2 | 11.2 | 2007 | 1.7 |
| 52 | 3DSS | 49 | 28 | 4435.7 | 11.0 | 2008 | 1.8 |
| 53 | 3ZKQ | 19 | 24 | 1745.4 | 10.9 | 2013 | 1.51 |
| 54 | 2NPT | 14 | 10 | 1292.8 | 10.8 | 2006 | 1.75 |
| 55 | 2A5D | 21 | 18 | 1941.3 | 10.8 | 2005 | 1.8 |
| 56 | 1NRJ | 23 | 26 | 2148.0 | 10.7 | 2003 | 1.7 |
| 57 | 2WY8 | 17 | 14 | 1600.4 | 10.6 | 2009 | 1.7 |
| 58 | 4DRI | 8 | 6 | 772.0 | 10.4 | 2012 | 1.45 |
| 59 | 2FCW | 15 | 6 | 1465.1 | 10.2 | 2005 | 1.26 |
| 60 | 2FOM | 34 | 32 | 3396.9 | 10.0 | 2006 | 1.5 |
| 61 | 3PRO | 24 | 33 | 2428.4 | 9.9 | 1998 | 1.8 |
| 62 | 4FBJ | 27 | 15 | 2740.3 | 9.9 | 2012 | 1.6 |
| 63 | 1SVD | 29 | 29 | 2997.1 | 9.7 | 2004 | 1.8 |
| 64 | 3GMO | 22 | 16 | 2321.4 | 9.5 | 2009 | 1.6 |
| 65 | 4G1Q | 49 | 39 | 5267.8 | 9.3 | 2012 | 1.51 |
| 66 | 2OZN | 15 | 6 | 1623.5 | 9.2 | 2007 | 1.6 |
| 67 | 3QN1 | 16 | 16 | 1743.1 | 9.2 | 2011 | 1.8 |
| 68 | 3WHT | 14 | 12 | 1542.8 | 9.1 | 2012 | 1.8 |
| 69 | 4IUC | 72 | 74 | 7988.7 | 9.0 | 2013 | 1.45 |
| 70 | 3ML1 | 56 | 56 | 6293.4 | 8.9 | 2010 | 1.6 |
| 71 | 3RNQ | 16 | 17 | 1854.8 | 8.6 | 2011 | 1.6 |
| 72 | 2NW2 | 38 | 49 | 4504.9 | 8.4 | 2006 | 1.4 |
| 73 | 4CRU | 33 | 27 | 3913.8 | 8.4 | 2014 | 1.64 |
| 74 | 2F91 | 15 | 17 | 1784.4 | 8.4 | 2005 | 1.2 |
| 75 | 4G7X | 13 | 21 | 1547.6 | 8.4 | 2012 | 1.44 |
| 76 | 4MBG | 61 | 81 | 7291.9 | 8.4 | 2013 | 1.74 |
| 77 | 1G8K | 32 | 33 | 3943.3 | 8.1 | 2000 | 1.64 |
| 78 | 1Z3E | 11 | 9 | 1356.4 | 8.1 | 2005 | 1.5 |
| 79 | 1ZLH | 17 | 19 | 2153.9 | 7.9 | 2005 | 1.7 |
| 80 | 3VZ9 | 26 | 20 | 3301.2 | 7.9 | 2012 | 1.03 |
| 81 | 3F1N | 13 | 15 | 1666.7 | 7.8 | 2008 | 1.479 |
| 82 | 2AQ2 | 11 | 21 | 1428.3 | 7.7 | 2005 | 1.8 |
| 83 | 3FPN | 12 | 21 | 1572.7 | 7.6 | 2009 | 1.8 |
| 84 | 2XFG | 17 | 27 | 2229.4 | 7.6 | 2010 | 1.679 |
| 85 | 2ZFD | 24 | 20 | 3170.0 | 7.6 | 2007 | 1.2 |
| 86 | 2V52 | 16 | 16 | 2140.8 | 7.5 | 2008 | 1.45 |
| 87 | 4CRW | 10 | 5 | 1376.4 | 7.3 | 2014 | 1.75 |
| 88 | 3MXN | 19 | 25 | 2621.6 | 7.2 | 2010 | 1.55 |
| 89 | 2NL9 | 15 | 3 | 2104.5 | 7.1 | 2006 | 1.55 |
| 90 | 3SBT | 11 | 14 | 1584.3 | 6.9 | 2011 | 1.799 |
| 91 | 4N9O | 9 | 21 | 1329.5 | 6.8 | 2013 | 1.5 |
| 92 | 2A9K | 12 | 15 | 1785.6 | 6.7 | 2005 | 1.73 |
| 93 | 3WN7 | 10 | 8 | 1497.7 | 6.7 | 2013 | 1.57 |
| 94 | 3VU9 | 16 | 23 | 2398.2 | 6.7 | 2012 | 1.75 |
| 95 | 3ZEU | 18 | 26 | 2758.2 | 6.5 | 2012 | 1.653 |
| 96 | 4AG1 | 12 | 19 | 1839.1 | 6.5 | 2012 | 1.4 |
| 97 | 4HDR | 23 | 33 | 3547.2 | 6.5 | 2012 | 1.45 |
| 98 | 2WWX | 22 | 27 | 3412.8 | 6.4 | 2009 | 1.5 |
| 99 | 2X83 | 7 | 15 | 1097.0 | 6.4 | 2010 | 1.7 |
| 100 | 2Z30 | 15 | 18 | 2377.5 | 6.3 | 2007 | 1.65 |
| 101 | 2ZSI | 16 | 16 | 2544.8 | 6.3 | 2008 | 1.8 |
| 102 | 3GJ3 | 6 | 13 | 956.4 | 6.3 | 2009 | 1.79 |
| 103 | 1E3D | 45 | 50 | 7217.4 | 6.2 | 2000 | 1.8 |
| 104 | 1PXV | 15 | 14 | 2421.2 | 6.2 | 2003 | 1.8 |
| 105 | 1R8S | 19 | 31 | 3083.9 | 6.2 | 2003 | 1.46 |
| 106 | 2P1M | 19 | 12 | 3221.2 | 5.9 | 2007 | 1.8 |
| 107 | 3CJS | 8 | 5 | 1374.3 | 5.8 | 2008 | 1.37 |
| 108 | 3KNB | 8 | 12 | 1381.5 | 5.8 | 2009 | 1.4 |
| 109 | 3P73 | 18 | 24 | 3120.4 | 5.8 | 2010 | 1.32 |
| 110 | 3KSE | 10 | 21 | 1754.8 | 5.7 | 2009 | 1.71 |
| 111 | 4A94 | 11 | 24 | 1935.9 | 5.7 | 2011 | 1.7 |
| 112 | 3DRA | 36 | 70 | 6363.8 | 5.7 | 2008 | 1.8 |
| 113 | 1MCV | 10 | 23 | 1778.3 | 5.6 | 2002 | 1.8 |
| 114 | 1T0P | 7 | 13 | 1268.1 | 5.5 | 2004 | 1.66 |
| 115 | 2QME | 10 | 14 | 1832.7 | 5.5 | 2007 | 1.75 |
| 116 | 1DPJ | 16 | 29 | 2983.9 | 5.4 | 1999 | 1.8 |
| 117 | 2R25 | 10 | 6 | 1873.5 | 5.3 | 2007 | 1.7 |
| 118 | 3SHG | 16 | 18 | 3003.9 | 5.3 | 2011 | 1.5 |
| 119 | 3H7H | 16 | 31 | 3007.1 | 5.3 | 2009 | 1.55 |
| 120 | 1DJ7 | 9 | 9 | 1692.5 | 5.3 | 1999 | 1.6 |
| 121 | 1MCT | 8 | 22 | 1526.2 | 5.2 | 1992 | 1.6 |
| 122 | 1PK1 | 5 | 10 | 968.0 | 5.2 | 2003 | 1.8 |
| 123 | 2WY3 | 11 | 13 | 2213.2 | 5.0 | 2009 | 1.8 |
| 124 | 1GO3 | 19 | 20 | 3823.2 | 5.0 | 2001 | 1.75 |
| 125 | 1JAT | 7 | 7 | 1475.5 | 4.7 | 2001 | 1.6 |
| 126 | 4LV5 | 9 | 14 | 1974.5 | 4.6 | 2013 | 1.7 |
| 127 | 1WQJ | 8 | 15 | 1759.0 | 4.5 | 2004 | 1.6 |
| 128 | 3BC1 | 9 | 9 | 2017.2 | 4.5 | 2007 | 1.8 |
| 129 | 4DH2 | 7 | 6 | 1631.2 | 4.3 | 2012 | 1.75 |
| 130 | 3H8K | 8 | 11 | 1994.5 | 4.0 | 2009 | 1.8 |
| 131 | 3KF6 | 8 | 20 | 2024.6 | 4.0 | 2009 | 1.65 |
| 132 | 2D7C | 5 | 13 | 1284.9 | 3.9 | 2005 | 1.75 |
| 133 | 3KTA | 21 | 26 | 5401.9 | 3.9 | 2009 | 1.627 |
| 134 | 3MMY | 11 | 21 | 3004.8 | 3.7 | 2010 | 1.65 |
| 135 | 3FPU | 10 | 27 | 2736.7 | 3.7 | 2009 | 1.76 |
| 136 | 2BLF | 10 | 31 | 2746.1 | 3.6 | 2005 | 1.8 |
| 137 | 2HQH | 4 | 12 | 1143.6 | 3.5 | 2006 | 1.8 |
| 138 | 2XPP | 6 | 7 | 1838.7 | 3.3 | 2010 | 1.74 |
| 139 | 3K90 | 3 | 10 | 936.8 | 3.2 | 2009 | 1.8 |
| 140 | 4GFT | 3 | 11 | 969.4 | 3.1 | 2012 | 1.6 |
| 141 | 4M6B | 6 | 11 | 1976.0 | 3.0 | 2013 | 1.78 |
| 142 | 1PQ1 | 6 | 7 | 2184.7 | 2.7 | 2003 | 1.65 |
| 143 | 2VN6 | 4 | 9 | 1485.6 | 2.7 | 2008 | 1.49 |
| 144 | 1P57 | 6 | 20 | 2477.2 | 2.4 | 2003 | 1.75 |
| 145 | 3K2M | 3 | 15 | 1242.4 | 2.4 | 2009 | 1.75 |
| 146 | 1RDQ | 5 | 19 | 2090.5 | 2.4 | 2003 | 1.26 |
| 147 | 2NNU | 3 | 2 | 1301.7 | 2.3 | 2006 | 1.59 |
| 148 | 2VU8 | 4 | 23 | 1776.5 | 2.3 | 2008 | 1.8 |
| 149 | 3IXS | 4 | 11 | 2049.6 | 2.0 | 2009 | 1.7 |
| 150 | 2BNU | 9 | 37 | 4862.9 | 1.9 | 2005 | 1.4 |
| 151 | 3P8B | 3 | 18 | 2481.0 | 1.2 | 2010 | 1.8 |
| 152 | 3DBO | 2 | 15 | 3495.8 | 0.6 | 2008 | 1.76 |
| 153 | 2YLE | 1 | 14 | 2036.3 | 0.5 | 2011 | 1.8 |

^1^Number of waters found at interfaces of crystal structure after being filtered by criteria described in Dataset Details

^2^Number of waters placed at the interfaces with the *Rosetta-ECO* method

^3^Interface size of complex AB computed using the calc_total_sasa function of Rosetta with a probe size of 1.4 Å. Interface size = SASA_A + SASA_B - SASA_AB.

*The 30 shaded entries were used for parameter training. All others used in reporting water recovery by *Rosetta-ECO* in Table 1 of the main text.

**2.) Docking Discrimination Datasets**

Two docking data sets were generated for binding energy calculation testing with various score functions: 1.) protein/ligand and 2.) protein/protein docking sets. For both, the goal was to generate ensembles of near-native and decoy binding conformations that were well-distributed in RMSD-space with respect to the experimental conformation. This was achieved through self-docking protocols in addition to perturbation of the native conformation in cases where near-native sampling via docking was poor.

For protein-ligand docking, 46 members of the Binding MOAD database[1] (those excluding ions or cofactors in the binding sites and enriched for cases in which water molecules are found at the interfaces) were used to evaluate improvements in differentiating native versus decoy docking poses (see Supporting Information for complete list). Ligands parameterized with AM1-BCC partial atomic charges[2] and locally docked to the native binding site with RosettaLigand[3]. For each ligand, 3000 decoys were generated by docking 30 alternative ligand conformations 100 times. Near-native conformations were generated using the “minimize” option in RosettaLigand[3] starting with the experimental structure. Finally, after generating an initial docking conformations with the default RosettaLigand energy function, the lower edge of the ΔG_bind_ vs RMSD to native energy distribution was selected as the final test set. This created a total of 6376 total docking conformations including both near-natives and decoys.

For protein-protein docking, 53 cases with a total of 59,738 docking conformations were generated with ZDock 3.0[4], followed by local optimization with RosettaDock[5], implementing the same protocol as in *dualOptE*[6]. This ZDock subset was selected for size/runtime concerns and was additionally truncated for cases with disulfides across the binding interface. For both protein-protein and protein-ligand datasets, there is continuous sampling in the RMSD dimension with respect to the native structure.

3.) **Protein-Ligand Docking Set (46 cases):**

6376 total models including both near-natives and decoys

1GPK, 1HNN, 1JLA, 1KE5, 1KZK, 1L2S, 1M2Z, 1N1M, 1N2J, 1N46, 1NAV, 1OF1, 1OF6, 1OPK, 1OWE, 1P62, 1PMN, 1Q1G, 1Q41, 1R55, 1S19, 1S3V, 1SQN, 1T40, 1T46, 1TOW, 1TT1, 1TZ8, 1U1C, 1U4D, 1UNL, 1UOU, 1V0P, 1VCJ, 1W2G, 1X8X, 1XM6, 1XOQ, 1Y6B, 1YV3, 1YVF, 1YWR, 1Z95, 2BM2, 2BR1, 2BSM

**4.) Protein-Protein Docking Set (53 cases):**

59,738 total models including both near-natives and decoys

1A2K, 1AHW, 1AKJ, 1AVX, 1BJ1, 1BUH, 1BVK, 1DFJ, 1E6E, 1EAW, 1EZU, 1F34, 1F51, 1FFW, 1FQ1, 1FSK, 1GLA, 1GPW, 1H1V, 1HCF, 1IB1, 1J2J, 1JMO, 1JZD, 1KKL, 1M10, 1NCA, 1NSN, 1OPH, 1QA9, 1R6Q, 1RV6, 1SYX, 1US7, 1XU1, 1YVB, 1ZHI, 2A5T, 2AJF, 2AYO, 2CFH, 2H7V, 2I9B, 2JEL, 2MTA, 2O3B, 2OOB, 2PCC, 2UUY, 2VIS, 3CPH, 3D5S, 7CEI

**5.) GOLD Docking Protocol / Dataset**

Ligand docking was carried out to the protein.mol2 files from the Astex Diverse Set available for download from the CCDC website (<https://www.ccdc.cam.ac.uk/support-and-resources/downloads/)>. Prior to docking, ligands and cofactors were assigned partial atomic charges using the AM1-BCC method in *Antechamber[7]*. Each ligand was then docked as described by Liebeschuetz, et al., using the ChemPLP score function with the standard genetic algorithm settings with default early termination parameters which halts the GA when the top three ligand poses are within 1.5 Å of each other[8]. To expand the number of decoys generated by GOLD, the starting point for GOLD docking was randomly perturbed by up to 6.0 Å from the center of geometry of the native ligand position, while maintaining the standard 6.0 Å search radius. The final set used for rescoring with Rosetta includes ~500 docking conformations for a 67-target subset of the Astex Diverse set, excluding the cases in which an ion coordinated the ligand in the binding pocket:

1U4D, 1XOZ, 1J3J, 1Q41, 1OF6, 1S3V, 1UOU, 1T46, 1IA1, 1N2J, 1OYT, 1K3U, 1GPK, 1M2Z, 1W1P, 1SQN, 1N46, 1R9O, 1Z95, 1V4S, 1OPK, 1L7F, 2BM2, 1JLA, 1N2V, 1TOW, 1N1M, 1U1C, 1OF1, 1SJ0, 1HWI, 1W2G, 1X8X, 1TZ8, 1YVF, 1S19, 1YV3, 1VCJ, 1NAV, 1SG0, 2BSM, 2BR1, 1UNL, 1LPZ, 1Q4G, 1V48, 1Q1G, 1P62, 1IG3, 1HNN, 1TT1, 1MEH, 1G9V, 1KZK, 1KE5, 1GM8, 1PMN, 1YWR, 1V0P, 1YGC, 1T9B, 1OWE, 1SQ5, 1HVY, 1T40, 1L2S

1. Benson ML, Smith RD, Khazanov NA, Dimcheff B, Beaver J, Dresslar P, et al. Binding MOAD, a high-quality protein-ligand database. Nucleic acids research. 2008;36(Database issue):D674-8. doi: 10.1093/nar/gkm911. PubMed PMID: 18055497; PubMed Central PMCID: PMCPMC2238910.

2. Jakalian A, Jack DB, Bayly CI. Fast, efficient generation of high-quality atomic charges. AM1-BCC model: II. Parameterization and validation. J Comput Chem. 2002;23(16):1623-41. doi: 10.1002/jcc.10128. PubMed PMID: WOS:000179022700012.

3. Meiler J, Baker D. ROSETTALIGAND: protein-small molecule docking with full side-chain flexibility. Proteins. 2006;65(3):538-48. doi: 10.1002/prot.21086. PubMed PMID: 16972285.

4. Pierce BG, Hourai Y, Weng Z. Accelerating protein docking in ZDOCK using an advanced 3D convolution library. PloS one. 2011;6(9):e24657. doi: 10.1371/journal.pone.0024657. PubMed PMID: 21949741; PubMed Central PMCID: PMC3176283.

5. Gray JJ, Moughon S, Wang C, Schueler-Furman O, Kuhlman B, Rohl CA, et al. Protein-protein docking with simultaneous optimization of rigid-body displacement and side-chain conformations. Journal of molecular biology. 2003;331(1):281-99. PubMed PMID: 12875852.

6. Park H, Bradley P, Greisen P, Liu Y, Mulligan VK, Kim DE, et al. Simultaneous Optimization of Biomolecular Energy Functions on Features from Small Molecules and Macromolecules. J Chem Theory Comput. 2016;12(12):6201-12. doi: 10.1021/acs.jctc.6b00819. PubMed PMID: WOS:000389866500044.

7. Wang J, Wang W, Kollman PA, Case DA. Automatic atom type and bond type perception in molecular mechanical calculations. J Mol Graph Model. 2006;25(2):247-60. doi: 10.1016/j.jmgm.2005.12.005. PubMed PMID: 16458552.

8. Liebeschuetz JW, Cole JC, Korb O. Pose prediction and virtual screening performance of GOLD scoring functions in a standardized test. Journal of computer-aided molecular design. 2012;26(6):737-48. doi: 10.1007/s10822-012-9551-4. PubMed PMID: 22371207.
