# Supplementary material for: Efficient consideration of coordinated water molecules improves computational protein-protein and protein-ligand docking discrimination
Source: PLoS Comput Biol. 2020 Sep 21;16(9):e1008103. doi: 10.1371/journal.pcbi.1008103 (PMC7529342; doi:10.1371/journal.pcbi.1008103)
Supplement: S3 Text — Includes information about CAPRI Target 47, details on the derivation of low-resolution statistical water potential, and more information on the force field parameter training tasks. (DOCX) [file pcbi.1008103.s024.docx]

**Details on *Rosetta-ECO* application to CAPRI Target 47:**

CAPRI target 47 was a blind challenge to model the protein-protein interface of the DNase domain of colicin E2 binding to the Im2 immunity protein, followed by prediction water molecule as the interface. We retroactively created our own models of this target using Rosetta homology modelling tools, then applied *Rosetta-ECO* to predict the interface water sites.

Homology models for the Im2-colicin E2 complex were build using RosettaCM with the Im2-colicin E9 complex (PDB ID 2WPT) as the sole template. The top five models, selected by total Rosetta score, were solvated using the *Rosetta-ECO* protocol described in this paper.

*Rosetta-ECO* was able to consistently predict the position of three of the 9 buried interface waters as described in Lensink et al. Our analysis indicated that water #8 in the set of buried waters is not coordinated by a polar atom of Arg 38.A as indicated in Figure 1 of the report, and thus does not meet the criteria of being a water that mediates the contact between the two protein binding components. Our top 5 models recall waters #2, #3, and #6 with relatively high precision (generally within 1.0 Å, and often closer to 0.5 Å). Visual analysis implies that these happen to be the same waters that are conserved in the modeling templates and are strongly coordinated by backbone polar groups.

For a more direct comparison to the participant submissions of the CAPRI challenge, we implement the f^W^(nat) and f^W^(un-nat) equations as described by Lensink et al. to quantify the percentage of accurately predicted interface waters and possible false positives based on the crystallographic data. Using the metrics described in the paper, we were able to identify 22 interface waters in the released crystal structure, not 23 as described, and used a water recovery tolerance of 2.0 Å as adopted for general model assessment.

Table S2 in Lensink et al., lists the model scores for those with 'excellent' quality for f^W^(nat) using a distance cutoff of 2.0 Å. Two of our 5 models would have placed within this classification with a cutoff of f^W^(nat)=0.500, as detailed in Table S5 below. The 'excellent' models of all participant-submitted models had a f^W^(nat) range of 0.522-0.913 and a f^W^(un-nat) range of 0.000-0.400. With relatively few false positives for this specific case, our f^W^(un-nat) scores are quite strong, while for models #1 and #4, ~50% of the waters placed by *Rosetta-ECO* are within 2.0 Å of a crystallographic water.

**Table S5. Water recovery details for top 5 Im2-colicin E2 models**

| Model # | # waters placed | Predicted waters within 2.0 Å of native | f^W^(nat) | f^W^(un-nat) |
| --- | --- | --- | --- | --- |
| 1 | 14 | 11 | 0.5000 | 0.136 |
| 2 | 12 | 8 | 0.364 | 0.182 |
| 3 | 10 | 7 | 0.318 | 0.136 |
| 4 | 13 | 11 | 0.500 | 0.091 |
| 5 | 12 | 8 | 0.364 | 0.182 |

Of our best models, model #4 has 13 water molecules placed at the interface of which only 2 were not with 2.0 Å of a crystallographic water, resulting in f^W^(nat) and f^W^(un-nat) of 0.500 and 0.091, respectively. Based off of numbers reported in the CAPRI challenge publication, this places our best effort in line with the top submitted models.

**Details on Derivation and Use of Statistical Potential:**

A total of 14,053,883 water/protein measurements from 6,342 structures of the Top8000 database were used to determine the probability of finding water molecules at particular distances and angles from 28 different protein polar groups. Probability matrices, P(d,θ), were generated by binning distance and angle measurements with bin intervals of 0.25 Å and 7.5°, respectively, followed by normalization and smoothing via convolution with a Gaussian kernel. The final potential for each polar group takes the form of –log( P(d,θ) / P(d,θ)ref ), where the reference is the distribution of water about a non-polar group (the β-carbon of alanine). The final potential maps, as illustrated in Fig S14, were flattened to zero beyond 4 Å and 90° to only include first solvation shell effects. Additionally, testing has shown that normalized potentials result in better recall of natives, thus all point water potential minimums were set to a value of -1.5, which roughly corresponds to the score awarded to a single hydrogen bond in the full-atom Rosetta energy function.

The initial placement of water molecules to be scored by the point water potential come from two sources. First, possible water sites about backbone polar atoms are obtained from the same statistics used to develop the point water potential. From the experimental data set, all waters within 3.1 Å of a backbone carbonyl or nitrogen group were clustered into discrete representations of the entire statistical distribution based on k-means clustering. Ultimately, positions of 77,798 water molecules about C=O groups in the data set were clustered down to the 10 most-probable solvation sites (illustrated in Figs S15 & S16), while only a single site is used for the NH backbone group.

Given that the backbone is fixed in most Rosetta protocols, a strategy of building possible water sites based on statistics from the PDB is very efficient and accurate. However, this strategy becomes less feasible when applied to identifying potential solvation sites about the polar groups of side chains. Given the large number of rotameric states available to most polar side chains, the statistical distribution of water sites becomes very disperse. To overcome this problem, we instead implement a method in which idealized water positions about polar groups, as defined by Yanover and Bradley[29], are accumulated for all rotamers available to each amino acid that is being considered for solvation. The resulting set of potential water sites is then reduced to only keep the average position of pairs of waters within 0.75 Å of each other that originate from two different residues, with the goal of obtaining possible water sites that could bridge the interaction between two side chains. The resulting positions are further culled by removing duplicate positions within 1.0 Å of each other. The final positions are then clustered with a 3.0 Å radius to form rotamer sets for each grouping.

Using the statistical point water potential to solvate a protein surface or interface involves a modified version of the standard Monte Carlo (MC) packing algorithm used in Rosetta. For each position on a protein to be solvated, clouds of solvation sites are built off a fixed backbone. Each collection of solvation sites is treated as a new residue composed of point water ‘rotamers’. During the packing simulation, a single rotamer is selected from the entire rotamer set including both point waters and side chains. The rotamer is applied to the pose, scored, then accepted or rejected based on the Metropolis criterion. Since a majority of the water rotamers are expected to score poorly, each water residue is assigned an extra ‘virtual’ state which is sampled 50% of the time a water rotamer is randomly selected to help convergence of the simulation.

Test simulations with the point water potential have shown that the most relevant water states were visited during low temperatures. Therefore, long simulations at a single low temperature (RT = 0.3 kcal/mol) were used as an alternative to the default simulated annealing protocol of Rosetta. Periodic temperature spikes at 100 K serve to scramble the overall conformation and allow for better convergence and reproducibility. For a packing simulation with a total number of rotamers equal to nrot, data is collected at the low temperature for 5*nrot MC steps, followed by a high temperature spike for nrot steps. Before data collection is resumed at the low temperature, a burn-in period of nrot steps is implemented. In total, data is collected for 50 cycles of temperature spiking.

During the packing simulation, the dwell time for each point water rotamer is recorded and ultimately normalize to the total number of low-temperature MC steps. Those positions with a dwell time below a specified cutoff are discarded and the remaining positions are clustered to dwell time-weighted centroid positions. A second cutoff is used to remove clusters with a cumulative dwell time below a certain threshold. The final centroid positions are then converted to full-atom water molecules. The water molecules, which sample positions in rotational space and again include a virtual state, are packed one final time to make use of the full Rosetta energy function, further discriminating between the true positive positions from the false.

A number of the Top8000 set structures overlap with our water recovery validation set. Although the Top8000 structures were primarily used to derive the low-resolution point water potential which undergoes a level of smoothing, we still tested water recovery on the subset of structure that were not used for parameter/potential training in any way. Removing the 44 structures from the Top8000 set that overlap with our validation set, left a test set of 79 structure with 1865 water positions. We recover 17.66% of these waters with a precision of 17.82%, compared to the recovery of 17.69% with a precision of 17.71% when applied to the full validation set of 123 structures with 2815 water positions. This indicates that there was no overfitting during the parameterization process.

**Description of Training Tasks and Results on Test Sets:**

Three different classes of training tasks were used in the parameterization of the *Rosetta-ICO* energy function, which were the same used for the development of the *REF2015* energy function. While the tasks are described in detail in the REF2015 paper (Park et al, 2016), they are summarized below.

Briefly, the three categories include: structure prediction, sequence design, and high-resolution structure recovery. For the structure prediction tests, two sets of monomeric protein folding energy landscapes are used to evaluate the ability of the new energy function to properly identify near-native structures from decoys. For sequence design, individual residues of protein monomers as well as residues at protein-protein and protein-ligand interfaces are mutated to all 20 amino acids, followed be re-optimization of the structure with a fixed backbone, and scoring with an entropy-weighted profile recovery metric. Additionally, correlation to mutational ΔΔG were computed as a further evaluation metric. Finally, for the high-resolution structure recovery test, atom-pair distributions are compared for structure refined with two rounds of the FastRelax protocol of Rosetta in Cartesian space using the target score functions.

For structure prediction tasks, we report the Boltzmann weight of near-native decoys and the percent of native structures recovered; for sequence design, we report the entropy-weighted native recovery except for ΔΔG which reports the Pearson correlation; and for the high-resolution structure recovery, we report the relative error. The results are presented in Table S6. For all tests except high-resolution recovery, higher values are indicative of improved performance. Overall we see similar performance between REF2015 and *Rosetta-ICO* on monomeric structure prediction and protein design tasks. Looking at the “percent success” metric, we see a modest improvement in decoy discrimination, from ~62% to 65%; in all other metrics the two score terms are comparable. Therefore, we believe this is a reasonable general-purpose energy function.

**Table S6. Performance of energy functions on training task test sets**

| Tasks | | *REF2015* | *Rosetta-ICO* |
| --- | --- | --- | --- |
| structure prediction | decoy discrimination, set 1 | 0.705 (57.1) | 0.728 (64.9) |
|  | decoy discrimination, set 2 | 0.781 (67.2) | 0.761 (65.6) |
| sequence design | protein monomer | 0.282 (47.0) | 0.290 (45.6) |
|  | protein-protein interface | 0.316 (51.0) | 0.314 (50.9) |
|  | protein-ligand interface | 0.425 (65.5) | 0.423 (59.2) |
|  | mutational ΔΔG | 0.743 (72.9) | 0.733 (71.3) |
| High-resolution structure recovery | atom-pair distribution | 0.00796 | 0.00949 |
