# Supplementary material for: Efficient consideration of coordinated water molecules improves computational protein-protein and protein-ligand docking discrimination
Source: PLoS Comput Biol. 2020 Sep 21;16(9):e1008103. doi: 10.1371/journal.pcbi.1008103 (PMC7529342; doi:10.1371/journal.pcbi.1008103)
Supplement: S1 Script — RosettaScripts XML file used for protein-protein / protein-ligand interface scoring with explicit water molecules (Rosetta-ECO). (DOCX) [file pcbi.1008103.s025.docx]

S1 XML Script.

**RosettaScripts XML file used for protein-protein / protein-ligand interface scoring with explicit water molecules (*Rosetta-ECO*):**

<ROSETTASCRIPTS>

<SCOREFXNS>

<ScoreFunction name="beta" weights="beta_nov16"/>

</SCOREFXNS>

<TASKOPERATIONS>

<RestrictToInterfaceVector name="interface" chain1_num="1" chain2_num="2" CB_dist_cutoff="10.0" nearby_atom_cutoff="5.5" vector_angle_cutoff="75.0" vector_dist_cutoff="9.0" include_all_water="1"/>

<ExtraRotamersGeneric name="extra_chi" ex1="1" ex2="1" extrachi_cutoff="0"/>

<RestrictToRepacking name="restrict"/>

</TASKOPERATIONS>

<MOVERS>

<ddG name="ddG" scorefxn="beta" chain_num="2" solvate="1" repack_bound="1" repack_unbound="1" solvate_rbmin="0" solvate_unbound="0" min_water_jump="1" task_operations="interface,extra_chi,restrict"/>

</MOVERS>

<PROTOCOLS>

<Add mover_name="ddG"/>

</PROTOCOLS>

</ROSETTASCRIPTS>
