# Supplementary material for: Efficient consideration of coordinated water molecules improves computational protein-protein and protein-ligand docking discrimination
Source: PLoS Comput Biol. 2020 Sep 21;16(9):e1008103. doi: 10.1371/journal.pcbi.1008103 (PMC7529342; doi:10.1371/journal.pcbi.1008103)
Supplement: Attachment — Submitted filename: reviewer_comments.pdf [file pcbi.1008103.s026.pdf]

Dear Editor-in-chief;

Enclosed is a resubmission of our manuscript, "*Efficient consideration of coordinated water molecules improves computational protein-protein and protein-ligand docking discrimination*." On the whole, we are happy to see that the reviewers found this work of interest and most of the issues were related to clarity of presentation. Consequently, our revised manuscript works on improving this clarity, rewording large portions of the text and equations, as well as adding a few explanatory supplemental figures.

Responses to individual reviewer comments are outlined below.

1. The CAPRI challenge included a water-placement target a few years ago. It would be interesting to see how these methods could perform on that target, and it would provide a clear metric of these methods against a large set of methods in the field. See https://www.ncbi.nlm.nih.gov/pmc/articles/PMC4582081/ for details; at CAPRI meetings, Lensink has even offered to provide the evaluation scripts as needed.

Homology models for the Im2-colicin E2 complex were built using RosettaCM with the Im2-colicin E9 complex (PDB ID 2WPT) as the sole template. The top five models, selected by total Rosetta score, were solvated using the same protocol described in the paper for the interface rotamer and experimental water recovery.

Rosetta-*ECO* was able to consistently predict the position of three of the 9 buried interface waters as described in Lensink et al., 2015 (Blind Prediction of Interfacial Water Positions in CAPRI). Our analysis indicated that water #8 in this set of buried waters is not coordinated by a polar atom of Arg 38.A as indicated in Figure 1 of the report, and thus does not meet the criteria of being a water that mediates the contact between the two protein binding components. Our top 5 models recall waters #2, #3, and #6 with relatively high precision (generally within 1.0 Å, and often closer to 0.5 Å). Visual analysis implies that these happen to be the same waters that are conserved in the modeling templates and are strongly coordinated by backbone polar groups.

For a more direct comparison to the submission to the CAPRI challenge, we tried to implement the fW(nat) and fW(un-nat) equations as described by Lensink et al., 2014. Using the metrics described in the paper, we were able to identify 22 interface waters, not 23 as described, and used a recovery tolerance of 2.0 Å as adopted for the model scoring.

**Model #**	**# waters placed**	**Predicted waters within 2.0 Å of native**	**f^w(nat)**	**f^w(un-nat)**
**1**	**14**	**11**	**0.500**	**0.136**
**2**	**12**	**8**	**0.364**	**0.182**
**3**	**10**	**7**	**0.318**	**0.136**
**4**	**13**	**11**	**0.500**	**0.091**
**5**	**12**	**8**	**0.364**	**0.182**

Table S2 in Lensink et al., 2015 lists the models scores for those with 'excellent' quality for f^w(nat) with a distance of 2.0 Å. Two of our 5 models would have placed within this classification with a cutoff of f^w(nat)=0.500. The 'excellent' models have a f^w(nat) range of 0.522 - 0.913 and a f^w(un-nat) range of 0.000 - 0.400. With relatively few false positives for this specific case, our f^w(un-nat) scores are quite strong, while for models #1 and #4, ~50% of the waters placed by Rosetta-ECO are within 2.0 Å of a crystallographic water.

Of our best models, model #4 had 13 water molecules placed at the interface of which only 2 were not within 2.0 Å of a crystallographic water, resulting in f^w(nat) & f^w(un-nat) of 0.500 and 0.136, respectively. Based off of numbers reported in the CAPRI challenge publication, this places our best efforts in the top 6% of submitted models.

2. Please add a couple more sentences to the main text describing the main hypotheses and concepts behind the two models.

Clarifying and additional statements have been made on lines 54, 65, and 72.

3. In Figure 1A-D, the coordinate system is not clear. What are we looking at here?

The figure is illustrating a planar cut of the solvation energy terms between two atoms (a donor N atom from a backbone N-H group and an acceptor O atom from a different backbone C=O group) with the x- and y-axes reflecting the relative position of the two atoms with respect to one another.

The axes of the plots have been altered to better indicate that movement along either axis represent movement of the interacting pair of atoms.

4. Binding energy: are the separated components repacked or relaxed? I think not from the methods, please specify (line 470). (Chaudhury found that not relaxing shows a clearer signal for docking; he called it "interface energy" to distinguish it from the relaxed-monomer case which is more like nature.) Also are waters in the separated components placed all over the monomers or just at the binding interface? It was nice that the method buries about the same number of waters at interfaces as Janin counted in crystals; can you also compare the waters at the separated interfaces and are they also like Janin or another reference of structured waters?

We did not try the Rosetta relax protocol in our ddG method development. However, in both the bound and unbound states, side chains that define the interface (as defined by Rosetta's "RestrictToInterfaceVector" operation) are allowed to pack and minimize. Thus, we are more inline with 'binding energy' calculations than the 'interface energies' of Chaudhury.

Clarifying statements have been made to section 5 of the methods to make it clear that waters are only added to the interface of the bound models, with re-solvation of the separated components limiting new water placement to the region that was defined as being part of the interface in the bound state.

Additionally, we have added sample XML scripts to show exactly how the binding energy scores were calculated.

Finally, the Janin paper only gives stats on solvated interfaces, not the separated components. However, in our tests, there's an average of ~2 more water molecules

at the combined interfaces in our unbound structures than in the bound structures (19 average waters in the bound state vs 21 average waters in the unbound state).

5. It seems like the docking is done with a rigid protein backbone. Please confirm and make sure that is clear in the paper. Or, if there is backbone motion, describe and explain its impact on the results.

Yes, all conformational sampling used to generate the docking datasets involved fixed protein backbones. A clarifying statement has been added to Section3 (Datasets) of the main text.

Methods questions:

6. Table 2: which rmsd measure is used? Lrmsd? Irmsd? Atom selection? Please define in methods.

The RMSD used in this study are CA-rmsd of the ligand with respect to native for protein-protein docking cases (where the ligand is second chain in the PDB of the "native"), and heavy atom rmsd of the ligand for protein-ligand docking cases. A clarifying statement has been added to the methods section.

7. Line 176: In the benchmark set, the 53 protein-protein interfaces are categorized as rigid, moderate or flexible backbone targets. Which were chosen? Is Rosetta-ICO and Rosetta-ECO more or less helpful on any of these categories?

The subset of the ZDock set was trimmed down from the full set for size (for speed concerns) and for cases with the potential to form disulfides across the interface. The categorization of rigid, medium, or difficult were not considered. Furthermore, given that we performed our tests using bound states with rigid backbones, and the 'difficulty' categorization refers to the difference between the bound state and apo conformations, such a difficulty rating does not apply to our use of these targets. However, our distribution of the three types is similar to that found in the full ZDock target set.

8. Line 177: As per line 466 and comment 4 above, use "interface energy"

This correction has been made.

9. Line 339: Add units on D0 and S0. I suggest replacing S0 with S0^2 in the formula so that both parameters can have dimensions of length. This would help make it physically interpretable.

Thank you for catching this. We modified S_0 to be S_0^2 in the equation for G. As for dimensions, all parameters should have dimensions of Ångstoms, which has been added to the text. $D_{0,len}$ = 0.5 Å, $D_{0,angle}$ = 4.33 Å, $S_{0,len}$ = sqrt(2.6) Å, $S_{0,angle}$ = sqrt(7.28) Å.

10. Line 335: Why was the form of G chosen? It seems like it might be very sharp at the minimum. Add a plot with the final values of D0 and S0 and units.

G was chosen to smoothly transition from 1 to 0 between D_0 and D_0+S_0. There is a piecewise aspect of the equation that was not properly described in the original text. We have now expanded the explanation of this equation to better describe the

precise behavior. The plot below shows what G(x) looks like for D_0=0.5 Å and S_0=sqrt(2.6)=1.61 Å.

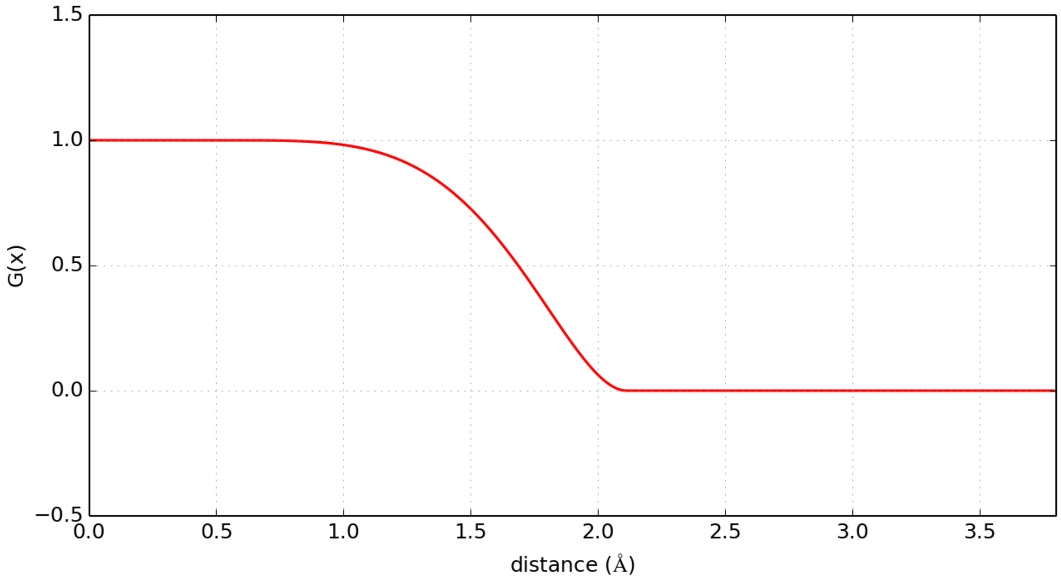

11. What is the reference energy for the lk-bridge term? As atoms move away, the energy goes to infinity! How is this handled?

The answer to #10 should resolve this question. G(x) switches to 0 at large distances (greater than D_0+S_0).

12. Line 340-341: I get how the second term targets for distance, but the first term seems to look to superpose the two water molecules. I guess 'angle' comes out of that, but I suggest rephrasing this sentence.

The first term is the overlap of lk_ball water positions that originate from the base atoms (which are used in the second term). It is beneficial for the theoretical water positions in the first term to fully overlap and thus receive the full value of Elk(i.j) since G(x) will equal 1. The second term indirectly targets for angle, by tethering the distance of the associated heavy atoms.

This has been clarified in the text.

13. Line 343: Please add a table of all the new parameters, to compare with the one in Alford et al and show the differences from the old set. Also, in the text, please give the nominal values for the new fitted parameters S0 and D0.

These tables have been added to the Supporting Information, including the DG_FREE, LJ_RADIUS, and LJ_WDEPTH values that were fit. Additionally, an explanation of the electrostatic scale factors that were used to adjust the partial atomic charges for the protein backbone and side chains is explained.

As mentioned in the response to #9, the values of S_0 and D_0 have been added to the main text.

14. Line 346: I'm not sure what 'quite reasonable' means. Change to state the fraction of time that a donor or acceptor violates its number of physically reasonable bonds.

We have removed this phrasing and expanded the statement to include how the Rosetta-ECO model includes explicit water molecules that ameliorate this potential over-coordination problem. Although it is theoretically possible for a donor or acceptors to violate the number of physically reasonable hydrogen bonds, we have not observed this in our limited test cases of natural proteins.

15. Line 348: citation doesn't seem right.

The citation has been updated to the following:

An Adaptive Dynamic Programming Algorithm for The Side Chain Placement Problem. A. Leaver-Fay, B. Kuhlman, and J.S. Snoeyink. In PSB 2005. 17-28.

16. Line 355: it took me a long time to figure out what was meant by treating the 'water asymmetrically'. Maybe add another example (in addition to the serine example) with the water atoms as an example. If I'm following correctly, the water will not even have a pseudo-torsion energy, correct?

To clarify, we modified the text to state that the potential was not symmetric about two water hydrogen atoms, as opposed to saying that "water was treated asymmetrically". And yes, water no longer has a pseudo-torsion energy.

17. Line 358: what is M? Please define with final value and units. Also I think the sign might be wrong here.

M describes the "softness" of the soft max, as M->0, it becomes more and more like a max, as it becomes larger, the transition becomes smoothed. We have added a clarifying statement in the text.

18. E_ref is a poor name for the energy of releasing a water to the bulk because this symbol is already used in Rosetta many times. How about E_bulk or E_water_release or E_WS for water/entropy…anything that is more directly tied to its meaning here.

This has been changed to E_bulk for the full-atom potential in the manuscript to be more descriptive of the physical effect of this term. We differentiated this from the analogous term in the lower-resolution point-water score: E_pwat_bulk.

The default values for these terms (1.221 kcal/mol and 2.709 kcal/mol) have been included in the text.

19. I wasn't sure the "lk" suffix is useful on the bridge energy term name. Sure, it's based on the Lazaridis-Karplus base model, but this is a whole new water approach. Is this just so it sorts in an alphabetical list near the other solvation terms? Maybe beyond the scope here, but maybe all solvation terms should be renamed E_solv_*, where * is lk or otherwise.

This is a good point. We opted for the _lk_ terminology however, as the term is balanced against and shares parameters with the lk model. Elk(i,j) in equation 1 is

actually the E_fa_sol term between atoms i and j. The description of this term has been expanded in the text.

20. Is the lk_ball model used in ICO or ECO?

Yes, the lk_ball model is used in both ICO and ECO (which is built on top of the ICO model). Devirtualized waters are treated as any other atom in the forcefield: they are capable of making interactions with bulk solvent (via the lk_ball models) as well as implicit bridging water interactions. A statement clarifying this has been added to the text.

21. Line 391, what is the capital W about? Should that be a script W to denote the set of waters placed?

W has been expanded in the text from W={wi} to W={w1…,wn} to indicate that the E_point-water term is summed over *all* point water positions and pairs.

22. Line 391, replace d() with a norm function as in the earlier equation.

This correction has been made.

23. Line 394 add a small picture; I presume the angle is between w, a and b?

Yes, the angle is between the water oxygen, the a polar atom on the amino acid side chain or backbone, and the base atom of the polar atom. In the case non-sp3 donor groups, the angle is between the hydrogen atom and donor atom. For acceptors and sp3-hybridized donors, the angle is between the polar atom and a connected base atom.

An example plot for the potential is illustrated in Figure S12 for a point water relative to the HD1/ND1 atoms of a histidine residue, and reproduced below, showing how the point water potential is a function of angle and distance with respect to polar amino acid atoms.

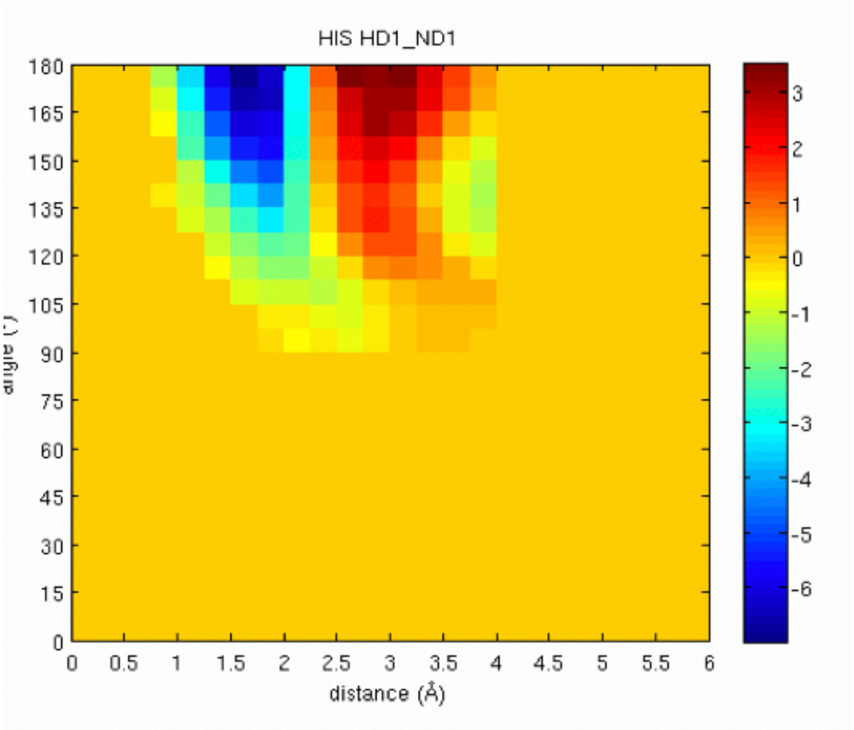

The second term of the point water potential provide a bonus if two point waters are within 2.7 Å of one another as plotted below:

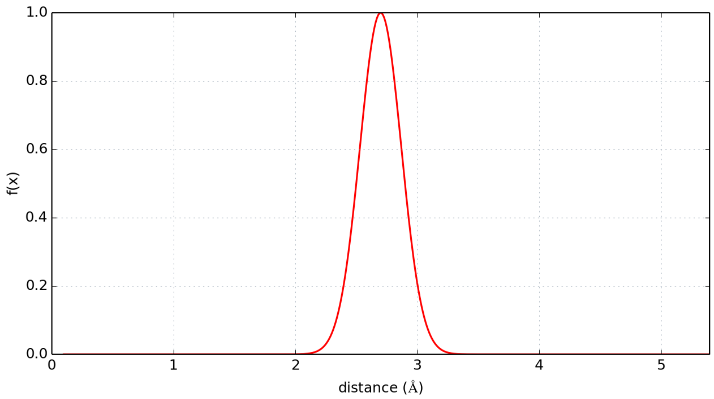

These have been added as a supplemental figure.

24. Line 397 give the final numbers for sigma and K with units.

The final values of K and sigma were added to the text (0.52 and 0.24, respectively).

Specifically, sigma = 1/sqrt(17.1) (the parameter pointwat_wat_bonus_width), while K = 0.52 (the parameter pointwat_wat_bonus)

25. Line 410, why not nitrogen backbone H-bonding sites too? Are side chain sites enumerated only from oxygens, not nitrogens?

Thank you for catching this: 1 'ideal' site from each backbone NH group is used during the point-water phase of Rosetta-*ECO*. This has been added to the text. For sidechains, the lk_ball positions for all polar atoms (N & O) are used for enumerating the possible point-water sites.

26. Paragraph starting line 411, I don't quite get the meaning and how this calculation is done. If rot_i1 clashes with rot_j1 but does not clash with rot_j2, what happens? When you detect overlaps in line 414 it sounds like it's about sc-sc overlaps, but line 417 seems to talk about w-sc overlaps too? Please clarify as this hash table approach seems to be a key to speeding the calculation relative to the older solvated rotamers approaches. What's in the hash table? Water positions?

Again, thank you for catching this. Where we previously mentioned the overlap of two different side chains, we meant the overlap of two different side chain *water* sites. This section has been reworded to make it more clear that we enumerate all ideal hydration sites for all rotamers (if designing, of all side chains) and use a hash table to efficiently determine all sites emanating from two different residues that "overlap" (come within 0.75 Å of one another). This has been clarified in the text.

27. For calculating 'dwell time,' is that only at the low temperature? After an equilibration period each time?

Yes, the 'dwell time' for each point water position is only recorded at the low temperature, following an equilibration period. This portion of the methods section has been clarified.

28. For the ECO monte carlo, please specify the MC move set and the fractional probability of calling each move type and the sizes of the moves (e.g. translations and rotations). The fold tree seems important too…do the waters move with one or both docking partners? Maybe all molecules (protein, ligand, and water) tie to a virtual origin point to move independently?

We have added text to clarify the MC routine is mostly unchanged for the ECO model, except that when a water rotamer is selected, there is a 50% chance that the 'virtual' state of the water molecule is sampled instead of the rotational state. A statement has also been added to clarify that a water rotamer is a rotational state about a fixed oxygen position.

As for the fold tree structure, the water molecules are connected via a rigid-body transformation (in Rosetta terminology, a "jump") to the nearest amino acid. This fold tree structure does not have an effect on packing, but does have an effect on minimization (they move with the "coordinating residue"). We have specified this explicitly as well in the text.

29. Line 466: Binding energies imply a transition from the unbound form of receptors and ligands to the bound form of the complex. The energy calculations described involve the energy of bound conformations of receptors and ligands as they form the binding complex, and would be better defined as the "interface" (or interaction) energy

As discussed in the response to #4, both bound and unbound states are solvated and sidechains are reoptimized ("packed") in our binding energy calculations, therefore they are more in line with traditional binding energy calculations than the 'interface' energies described by Chaudhury.

Grammatical edits

We have made these changes.

Reviewer #2: The manuscript by Pavlovicz et al. focuses on prediction of water molecules in protein-protein and protein-ligand complexes by implementing new methods in the Rosetta program. An impressive and original feature of the approach is that water positions and side chain rotamers are sampled at the same time, whereas most previous approaches have used a rigid protein. The authors also evaluate the performance of predictions of protein-protein and protein-ligand complexes. The research topic is relevant and important – waters are important for molecular recognition and accurate positioning of water should improve the performance of scoring functions. The results are mixed. In some cases, an improvement is observed with the waters included, in other cases it has no effect. I think that further analysis of the results is needed. I recommend that the following changes are made to the manuscript:

(1) Page 3: The authors write that their approach yields “superior results” at the end of the introduction. Compared to what?

This was meant with respect to predictions that involved no treatment of ordered water molecules. We have rephrased this in the manuscript resubmission.

(2) Page 4: I was surprised to read that only 17.7% of the native water molecules were predicted by the method for protein-protein interfaces. With previous methods (e.g. https://journals.plos.org/plosone/article?id=10.1371/journal.pone.0032036, https://pubs.acs.org/doi/10.1021/ci200150p) that are likely as fast or faster than the presented one, 76-97% of waters in crystal structures are predicted. These studies focus on proteins (not protein-protein complexes) and likely use rigid side chains, but can that explain the substantially lower performance of the method presented in this work? Or is there some other difference in the assessments (I think there might be some)? I recommend that the authors validate their method in the same way as these previous studies (e.g. with static side chains and same cutoffs) and comment on potential differences in the results. This analysis should not only include protein-protein complexes, but also protein-ligand complexes. This will give the reader a better idea of the performance.

There are multiple reasons for seemingly low 17.7% native recovery number reported in the paper:

1. **We are characterizing protein/protein rather than protein/small molecule datasets. The search space of potential waters is significantly higher than in the small molecule cases generally explored by WaterDock and AcquaAlta.**
2. **We are using very strict criteria for recovered waters. We only consider a water recovered if it is within 0.5 Å of native and has the native hydrogen bonding geometry.**
3. **Our approach (by design) errs on the side of underpredicting waters. We are most concerned with predicting waters positions that are not properly handled by our implicit model, thus, many waters (e.g., second- and third-shell waters, or waters at the periphery of interfaces that might be weakly coordinated) are not predicted by our approach.**

Our head-to-head benchmarks with another prediction tool (3D-RISM) shows that our recovery numbers are not so far off from this well-established tool.

Finally, we would like to re-stress that what we are doing with our placement algorithm, *simultaneous* prediction of side chain orientations with water placement, makes our method unique. Our method was specifically developed with this intention, meaning that alternate methods would have been explored and likely implemented if the intent was to work on static structures. Thus any direct comparison for water recovery against alternate tools (which were all developed to be applied to static structures) artificially handicaps our comparative performance.

Given this, we re-ran recovery tests with a more strictly-defined PPI water set where we excluded 'exposed' waters (this with fewer than 15 CA neighbors) and are coordinated by at least 2+ backbone/side chain atoms. With a 2.0 Å recovery tolerance (a cutoff used in a CAPRI challenge for water modeling), we achieve a 42.1% recovery with a 20% precision.

Text has been added to discussion to clarify these points.

(3) Page 7: The authors compare their results to 3D-RISM, which gives better results, but is much slower (20-fold). However, there are many very fast methods too, which have not been mentioned (see previous comment). The relative performance of the methods and their advantages/disadvantages should also be mentioned in the discussion.

This is correct, and we have added citations to the WaterMap and AcquaAlta methods (and mentioned that there are faster methods when discussing 3D-RISM). But the main point is that of the previous response, what we are doing is unique compared to these other approaches, simultaneous prediction of water sites and surrounding side chain conformations.

(4) Page 9: The improvement of docking performance for Rosetta-ECO is very encouraging. However, the low prediction accuracy for waters (page 4) makes me question if the results are "right for the right reason". The authors should quantify if the top-ranked complexes for which an improvement is observed also has accurately placed waters compared those observed in the crystal structure.

Hopefully our response to #2 provides more confidence that our accuracy is not as dire as the 17.7% value suggests. When it comes to direct comparison of waters for the protein-protein docking case, this is very difficult due to the relatively lower resolution of the structures in the ZDock set. Of the subset we used for our test, the average resolution is worse than 2.5 Å (ranging from 1.15 to 3.50 Å), at which we would expect limited accuracy in water placement (our water recovery statistics used PPI structures that were 1.80 Å or better with an average of 1.6 Å). The case in Figure 2, at 2.3Å resolution, does not show many water molecules at the interface; of the 4 interface waters in 1E6E, *ECO* recovers 1 of these (at a distance of 0.4 Å).

As for the protein-ligand set, these structures are generally of higher resolution, and therefore have more reliable experimental water positions. In addition to the structure for 1X8X that we detail in the response to #5, we more closely analyze two other cases in which the application of Rosetta-ECO improved the discrimination score (caused the near-native binding energies to improve with respect to calculations performed without explicit water molecules).

For the first of these structures, PDB ID 1U4D, *Rosetta-ECO* predicts 3 waters within 3.5 Å of both the ligand and the protein, as illustrated in Panel A of the figure below.

Two of these waters recover experimental water positions within 0.8 Å (Panel B) and 1.0 Å (Panel C). The closest experimental water to the final predicted position is 2.3 Å away.

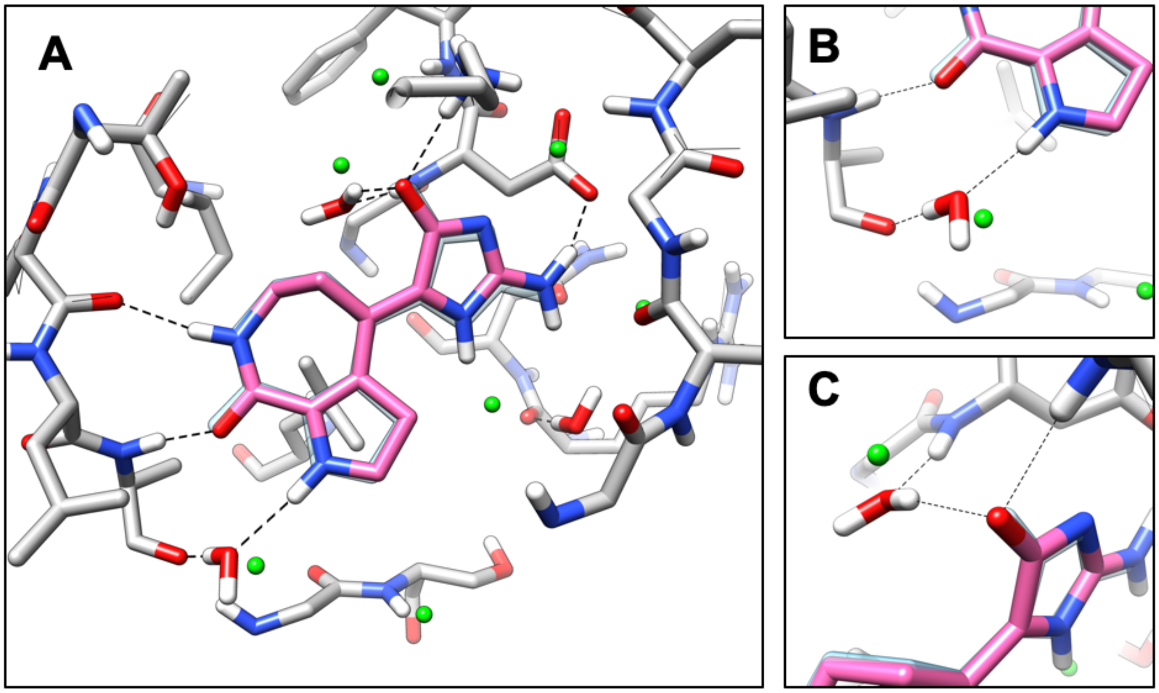

Figure. 1U4D binding mode with *Rosetta-ECO* model. The near native *Rosetta-ECO* model is in thicker stick representation with full-atom water molecules and the ligand depicted in pink. The experimental ligand position is in transparent blue, water oxygen positions as green spheres, and native side chains are in black wire representation. If the native ligand or side chain positions cannot be seen, it is because they are obscured by the Rosetta model. Panel A highlights the overall binding pocket, while panels B and C focus on recovered water positions.

For the other case we analyzed more closely, PDB 1N2J, *Rosetta-ECO* predicts 4 water molecules at the protein/ligand interface. Two of these directly bridge interactions between the ligand and protein, while the remaining two form a two-water bridge between the ligand and protein. All of these modeled waters come within 0.83 Å of an experimental water position. Panel B in the figure below highlights the interactions between the ligand and the two-water bridge that then interacts with the receptor. These two waters are predicted within 0.83 and 0.39 Å from experimental water positions. The accurately predicted bridging water molecules highlighted in Panels C and D come within 0.75 and 0.81 Å of experimental water positions.

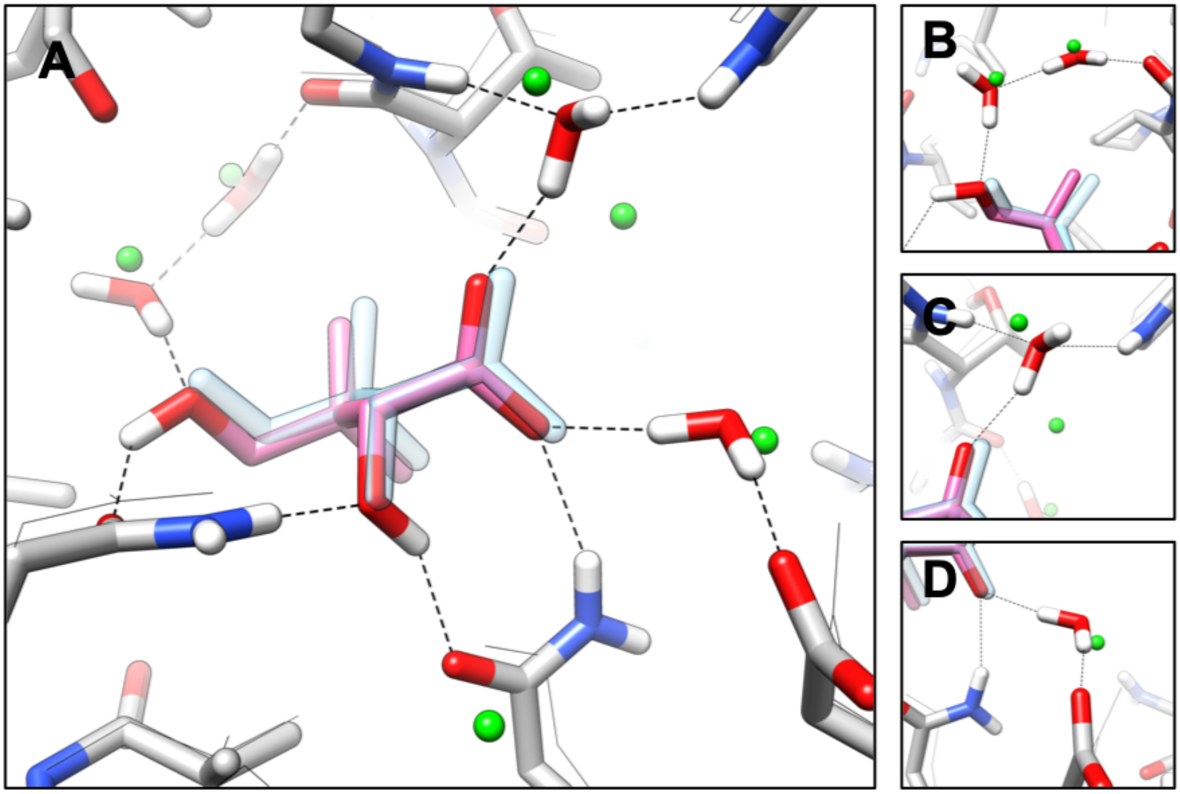

Figure. 1N2J binding mode with *Rosetta-ECO* model. The near native *Rosetta-ECO* model is in thicker stick representation with full-atom water molecules and the ligand depicted in pink. The experimental ligand position is in transparent blue, water oxygen positions as green spheres, and native side chains are in black wire representation. If the native ligand or side chain positions cannot be seen, it is because they are obscured by the Rosetta model. Panel A highlights the overall binding pocket, while panels B-D focus on recovered water positions.

A subset of the sample recovered water coordinations have been added to Figure 3 of the manuscript.

(5) Related to the previous question, in Figure 3C-D: Can you compare to the crystal structure water positions and side chain conformations in this case? How many of the water predictions are confirmed? Inspection of 1X8X revealed a phosphate close to the ligand.

It can be difficult to cleanly depict the binding mode with all of the side chains, but we are providing a view similar to those provided in our response to #4 where the *Rosetta-ECO* model is displayed as thicker sticks while the experimental structure is depicted as black wires. Most of these wires are not visible, indicating a very close overlay of the native structure and the model. In fact, the only incorrectly modeled side chain is for the ARG residue that coordinates the sulfate ion in the crystal structure.

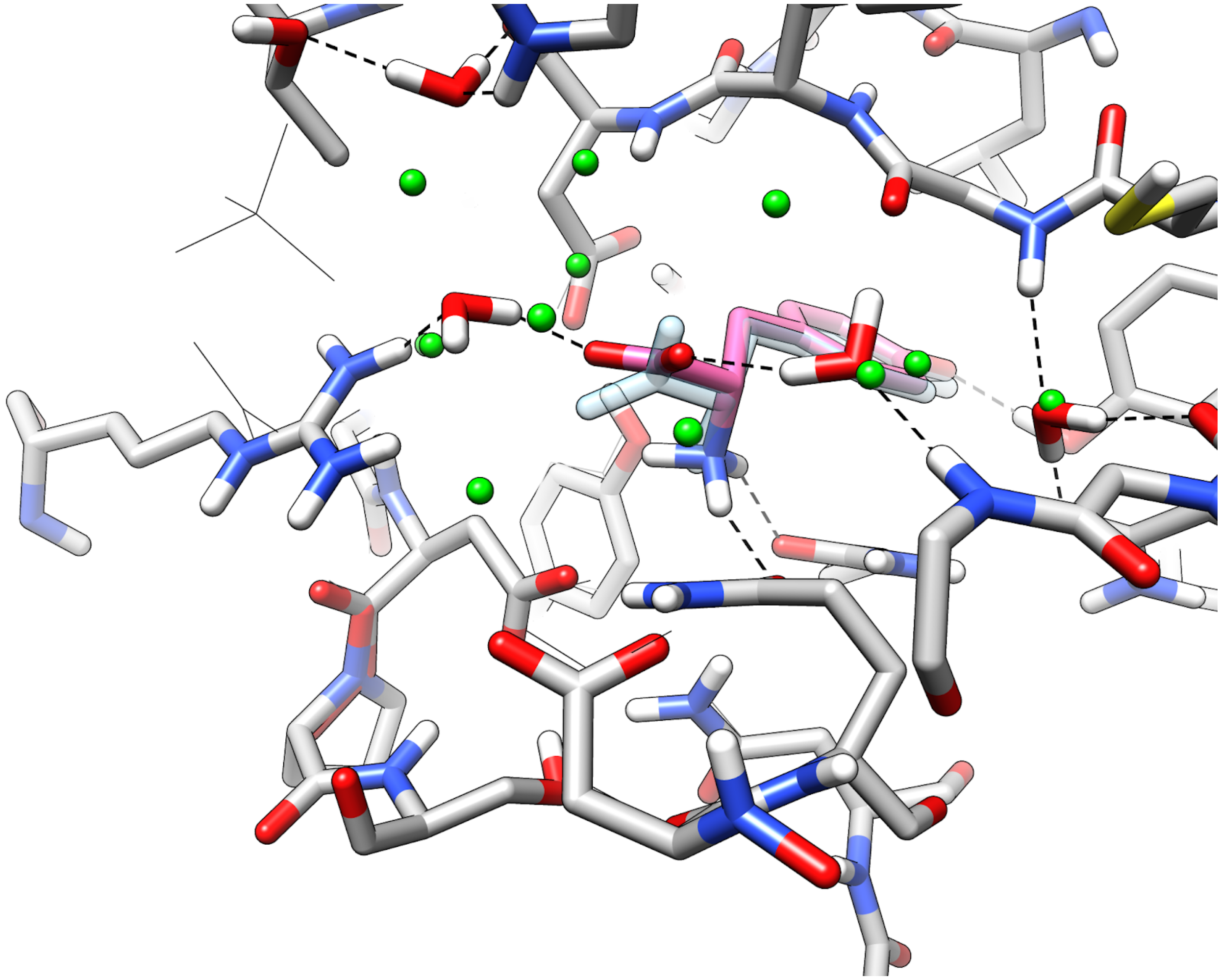

As for the sulfate ion, it is not directly interacting with the ligand, but is interacting with the ARG side chain that is slightly different than the input pose. The lack of the ion in our model is likely affected the incorrect side chain prediction.

Within the binding pocket, *Rosetta-ECO* predicts 3 water molecules within 3.5 Å of both protein and ligand. At most, these predicted water positions are 1.6 Å away from one of the crystallographic waters. The figure below highlights the interactions of the predicted water positions. Panel A focuses on the water molecule that is deepest in the pocket, which is recovered within 0.25 Å of a crystallographic water. This water does not hydrogen bond with the ligand, but does help shape the pocket. Panel B highlights a water that bridges the interaction between the carboxy terminus of the tryptophan ligand and the backbone of G198 (as shown in the foreground of Figure 3C). This water recovers a native position within 0.93 Å. The final water bridges the carboxy terminus of the ligand and the ARG side chain mentioned above. While the presence of the sulfate ion in the crystal structure likely disrupts the local water environment, this predicted water is still within 1.6 Å of one of experimental water sites.

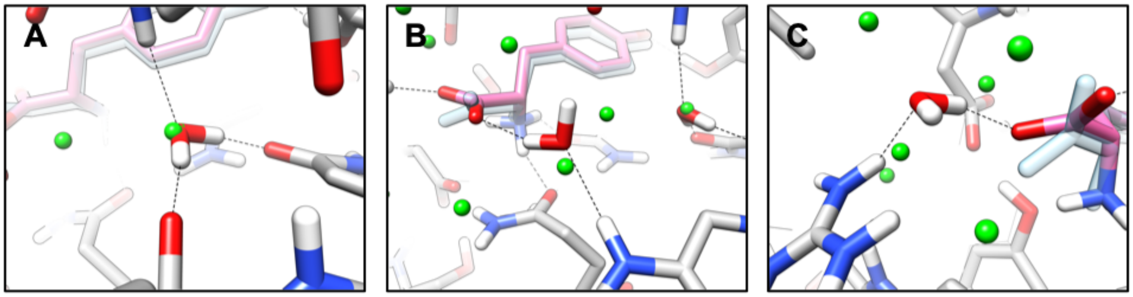
A
B
C
