# Supplementary material for: Efficient consideration of coordinated water molecules improves computational protein-protein and protein-ligand docking discrimination
Source: PLoS Comput Biol. 2020 Sep 21;16(9):e1008103. doi: 10.1371/journal.pcbi.1008103 (PMC7529342; doi:10.1371/journal.pcbi.1008103)
Supplement: Attachment — Submitted filename: reviewer_comments_2.pdf [file pcbi.1008103.s027.pdf]

Dear Editor-in-chief;

Enclosed is the second resubmission of our manuscript, "*Efficient consideration of coordinated water molecules improves computational protein-protein and protein-ligand docking discrimination*." We are happy to see that the reviewers found the initial revisions to be largely satisfactory and are in favor of allowing this new method to be published in your journal. In response to reviewer comments, we have: a) added additional information comparing our approach to alternate methods (WaterMap and WaterDock) and b) added a brief description of the performance of *Rosetta-ECO* on CAPRI Target 47.

Responses to individual reviewer comments are outlined below.

Reviewer #1: Thank you for the CAPRI target analysis. This is quite nice. I think you should include it in the paper, maybe even summarize the result in the abstract, because it places your work within the context of the field. At a minimum, put the text in your reviewer response in the Supplement.

We have added a brief summation of the CAPRI results in the Results section, while pointing to a more expanded examination of the results in the Supplement.

Reviewer #2: The authors have addressed most of my questions, and only a minor addition is required.

I am puzzled about why the authors think that comparison to simplified methods (e.g. WaterDock, WaterMap, AcquaAlta) is not so relevant. I think the authors should extend the clarifying section about this in the discussion and explicitly mention the results (e.g. % reproduced water) achieved by such methods. The difference is interesting for the reader and may influence the choice of method to apply in applications. The authors could provide some advice on this also based on the results.

As for comparing Rosetta-ECO to other methods, we do think this is a valid request, which is why we benchmarked against 3D-RISM, a well-regarded water prediction tool, to give a direct comparison against our method using exactly the same datasets and analysis scripts. We feel that this is sufficient to provide the general audience an understanding of where our new method fits into the landscape of water prediction methods. Ultimately, this paper is presenting a new method for water prediction that fits into the framework of the Rosetta software suite of protein design tools, providing the unique ability to predict water positions along with side chain conformations. While we have provided comparison to 3D-RISM for context, we feel that providing a full benchmark against all other water prediction tools (some of which require a costly license) is outside the scope of this Methods paper. To specifically address this concern we have expanded the text in the manuscript to give a more precise description of the performance of WaterDock and WaterMap on the task of predicting crystallographic water molecules, as determined in some published studies on alternate (but similar) datasets.

As for AcquaAlta, we have decided to remove the reference to this method based on a communication with the corresponding author of the paper, Martin Smiesko, who pointed out that their web server for the method is no longer functional and that the software is no longer supported. To our knowledge, the web server was the only way to access the method; a software download was never available.

From Martin Smiesko:

"The automatic processing of user queries on our server is no longer functional due to big changes in security measures that our computer/network support service had to deploy in fight against attacks. I will try to have a look if I could still somehow process your queries manually and send them back to you. Unfortunately this will require some trial and error, which might be not too straightforward quite time consuming. As I understand you would need this comparison rather urgently for the review of your paper.

Unfortunately, Acqua Alta had the same fate like many other PhD projects... After the student left, package was maintained for some time but then later got forgotten :("

Sincerely,
Ryan Pavlovicz and Frank DiMaio
